# Phytochemicals in Ruminant Diets: Mechanistic Insights, Product Quality Enhancement, and Pathways to Sustainable Milk and Meat Production—Invited Review

**DOI:** 10.3390/ani16030425

**Published:** 2026-01-29

**Authors:** Hasitha Priyashantha, Imasha S. Jayathissa, Janak K. Vidanarachchi, Shishanthi Jayarathna, Cletos Mapiye, Aristide Maggiolino, Eric N. Ponnampalam

**Affiliations:** 1 Department of Animal Science, Faculty of Agriculture, University of Ruhuna, Mapalana, Kamburupitiya 81100, Sri Lanka; shishanthij@agri.ruh.ac.lk; 2 Department of Animal Science, Faculty of Agriculture, University of Peradeniya, Peradeniya 20400, Sri Lanka; imashasj99@gmail.com (I.S.J.); janakvid@agri.pdn.ac.lk (J.K.V.); 3 Department of Animal Sciences, Faculty of AgriSciences, Stellenbosch University, Private Bag X1, Matieland 7602, South Africa; cmapiye@sun.ac.za; 4 Department of Veterinary Medicine, University of Bari A. Moro, Valenzano, 70010 Bari, Italy; aristide.maggiolino@uniba.it; 5 School of Agriculture, Food and Ecosystem Sciences, Faculty of Science, The University of Melbourne, Parkville, VIC 3010, Australia

**Keywords:** ruminant nutrition, sustainable livestock production, milk and meat quality, methane reduction, rumen fermentation

## Abstract

Dietary phytochemicals, primarily derived from grasses, legumes, and agro-industrial byproducts of plant origin, encompass distinct chemical classes such as polyphenols (including tannins, flavonoids, and other polyphenol compounds), saponins, organosulfur compounds, and essential oils (largely composed of terpenoids and phenylpropanoids). These compounds can function as rumen modifiers, antimethanogenic agents, anthelmintics, growth promoters, stress mitigators, and biopreservatives in ruminant production systems. Thus, they improve feed efficiency, nutrient utilization, and nitrogen retention while mitigating greenhouse gas emissions. In dairy systems specifically, phytogenic feedstuffs enhance milk yield and composition by enriching conjugated linoleic acids (CLAs), omega-3 fatty acids, and antioxidant compounds, leading to superior nutritional and oxidative stability. In meat production systems, they improve tenderness, flavor and shelf life through reduced oxidation and enhanced muscle metabolism. Despite these benefits, dose optimization, bio-efficacy, and species-specific responses remain critical research priorities. Use of phytogenic-based feeding strategies aligns with global sustainability goals by reducing reliance on feed additives, promoting environmentally resilient and circular food systems. This review synthesizes emerging evidence on the mechanisms, production outcomes, and functional benefits of dietary phytochemicals, providing a scientific framework for their strategic application in sustainable ruminant milk and meat production.

## Simple Summary

Ruminant animals such as cows, goats, and sheep play an important role in providing milk and meat for people around the world. Today, farmers and scientists are looking for natural ways to improve animal health, increase productivity, and reduce environmental problems that are often linked with livestock production systems. Many plants contain phytochemicals that can support these goals. When animals eat herbs, spices, grasses or legumes rich in phytochemicals, their digestion may become efficient, helping them get more nutrients from the same feed. These natural bioactive compounds can also reduce oxidative stress, methane production and nitrogenous emissions in livestock systems. As a result, animals may produce nutritious milk and meat with healthier fats, more antioxidants, and better flavor and shelf life with minimal impact on the environment. Overall, dietary phytochemicals offer a promising sustainable way to improve milk and meat production and quality while supporting animal health and reducing environmental impact.

## 1. Introduction

Ruminant production is crucial in addressing the nutritional needs of a rapidly growing global population, which is projected to reach 9.7 billion by 2050. These livestock food production systems contribute significantly to food security, providing approximately 16% of the world’s protein intake and 8% of total energy consumption through milk and meat products [1]. In developing regions, ruminant-derived foods are essential for combating malnutrition, supplying bioavailable micronutrients such as iron, zinc, and vitamin B12, often deficient in plant-based diets. van Vliet et al. [2] highlighted that grass-fed ruminant products contain health-promoting phytonutrients in quantities comparable to those in fruits and vegetables, enhancing their value in human nutrition. Moreover, ruminants’ unique digestive physiology enables them to convert fibrous, low-quality phytogenic forages and agro-industrial byproducts of plant origin into high-value edible products, promoting circular bioeconomy in marginal lands unsuitable for crop cultivation [3]. This capability supports livelihoods in pastoral systems and aligns with sustainable development goal target 2.4, which focuses on ensuring sustainable food production systems and resilient agricultural practices. This is critical since ruminant supply chains are under growing scrutiny because they account for a substantial share of agricultural greenhouse gas emissions, particularly enteric methane, and thus are central to global mitigation roadmaps for livestock agrifood systems [4,5,6].

The integration of dietary phytochemicals has shifted from being viewed as an antinutritional factor to considering valuable bioactive compounds for enhancing ruminant production systems. Bioactive phytochemicals, including polyphenols, essential oils, saponins, and organosulfur compounds, are abundant in herbs, spices, legumes, grasses, tree foliage and agro-industrial byproducts of plant origin [7]. Their roles extend to modulating rumen microbiota, reducing enteric methane emissions, improving nutrient utilization, and collectively boosting animal health and product quality [3,8,9]. In milk production, phytochemicals have been suggested to influence fatty acid profiles and oxidative stability, while in meat, they may enhance tenderness and shelf life by mitigating lipid, protein and myoglobin oxidation [10]. Beck & Gregorini [11] emphasized that diverse phytochemical-rich diets reduce environmental impacts by lowering greenhouse gas emissions from grazing animals. This evolution is driven by global challenges such as antimicrobial resistance, climate change, and consumer demand for natural, sustainable foods [12,13]. Consistently, a growing body of literature highlights phytochemical-rich feedstuffs, rather than isolated extracts, as promising natural alternatives to antibiotic growth promoters and ionophore-based rumen modifiers in ruminant production, owing to their synergistic matrices of bioactive compounds that support host health, immune function, and gut integrity [3,14,15,16].

The effects of dietary phytochemicals on feed intake, animal performance, and the quality of ruminant milk and meat vary considerably depending on various phytochemical (i.e., dosage, type, source, chemical structure, delivery method), dietary, microbial and host animal factors [17]. Delivery methods of phytochemicals in ruminants are often overlooked and receive little attention. Phytochemical delivery in ruminants involves providing raw or processed phytogenic feed individually or within complete diets, incorporating phytogenic extracts into total mixed rations, supplements or water, and administering phytogenic extracts via drenching. Drenching of phytogenic extracts is often combined with slow-release systems such as encapsulation and nanotechnology to control release and enhance bioavailability. Phytogenic feedstuffs are raw or partially processed plant materials that supply both nutrients and phytochemicals in animal diets, while phytogenic extracts are concentrated products derived from extraction of specific bioactive phytochemical compounds. While phytogenic feedstuffs have variable composition and lower phytochemical content than phytogenic extracts, they are widely available, cost effective and provide synergistic effects from multiple compounds. Hence, phytogenic feedstuffs could be more suitable for livestock systems that are seeking cost-effective, natural additives with broad benefits. Moreover, phytogenic extracts could be ideal for systems aiming for more efficient and consistent animal performance and product quality enhancements.

On the one hand, a recent comprehensive systematic review by Mapiye et al. [18] reported that dietary phytochemical extracts show no clear effect on ruminant performance, with limited understanding of their mechanisms and transfer efficiency. Nonetheless, essential oils and polyphenol extracts appear promising for enhancing production, oxidative stability, and health attributes of ruminant-derived foods. On the other hand, moderate inclusion of phytogenic feedstuffs seem to improve animal growth rates, milk yield, and meat quality by optimizing rumen fermentation and biohydrogenation processes systems [19,20,21,22]. However, responses remain heterogeneous across studies, due to variation in application conditions. In addition, comparatively few contributions provide an integrated, farm-to-fork perspective that simultaneously links dietary phytochemicals with mechanistic rumen responses, animal performance, product composition, health value, shelf life, consumer acceptability and system-level sustainability indicators. The current review, therefore, explores the implications of dietary phytochemicals on ruminant performance, milk and meat quality, and overall system sustainability, synthesizing recent evidence to identify opportunities for innovation. By focusing on mechanisms, regional perspectives, and future directions, it aims to guide research and adoption in diverse contexts. In an era of heightened environmental scrutiny, dietary phytochemicals offer a natural, cost-effective alternative to feed additives, reducing feeding expenses, enhancing productivity, and adding value to ruminant products, thereby supporting the long-term viability of global livestock systems.

## 2. Review Methodology

This review synthesizes current scientific understanding of dietary phytochemicals in ruminant nutrition, focusing on their mechanistic roles, impacts on milk and meat quality, and contributions to sustainability. Literature searches were conducted in Web of Science and Scopus databases between March and September 2025, using combinations of keywords such as “dietary phytochemicals”, “ruminants”, “rumen microbiota”, “milk quality”, “meat quality”, “methane reduction”, “sustainability”, and “feed efficiency”. Studies were screened for relevance based on in vivo or integrated experimental evidence related to ruminant milk and meat production, product composition, and environmental outcomes. Non-ruminant species, in vitro-only trials, and human dietary interventions were excluded. The included studies were critically appraised for methodological rigor, experimental design clarity, and relevance to sustainability metrics. Evidence was organized thematically, linking biochemical mechanisms to production performance, product quality, and environmental implications. This approach provides an integrative synthesis of emerging research trends while identifying gaps in bioavailability assessment, dosage optimization, and long-term validation under diverse production systems.

## 3. Sources and Properties of Dietary Phytochemicals for Ruminants

Dietary phytochemicals in ruminant systems are primarily supplied by feedstuffs such as grasses, legumes, grasses, shrubs, tree foliage and agro-industrial byproducts of plant origin [23]. These compounds, belonging to chemical classes including alkaloids, sulfur-containing compounds, saponins, essential oils and polyphenols. Table 1 presents key dietary phytochemical sources, incorporating examples relevant to milk and meat production, with key bioactive properties and references from recent literature.

### 3.1. Grazed and Conserved Grasses

In most ruminant systems, the main entry point of phytochemicals is grazed or conserved forage, including temperate and tropical grasses, forbs and mixed swards that naturally contain polyphenol compounds, terpenes and carotenoids [2,43]. Diverse pasture mixtures and rational grazing management can markedly increase the intake of bioactive plant secondary metabolites, thereby enhancing the antioxidant protection degree and polyphenol content of milk and meat while simultaneously improving pasture utilization [11,43,44]. In extensive grass-based systems, these forage-borne phytochemicals are key drivers of the distinct volatile and nutraceutical profiles observed in grass-fed products compared with concentrate-based diets [19,45].

Common grass species utilized in ruminant nutrition contain diverse bioactive phytochemicals with demonstrated functional properties. Perennial ryegrass (*Lolium perenne*) accumulates flavonoids and phenolic acids that contribute to antioxidant activity in grazing systems [2,43]. Tall fescue (*Festuca arundinacea*), despite concerns about endophyte-associated alkaloids in certain cultivars, provides condensed tannins and polyphenol compounds when managed appropriately [46]. Orchardgrass (*Dactylis glomerata*) contains moderate levels of water-soluble carbohydrates and polyphenols that influence rumen fermentation patterns [43]. In tropical systems, guinea grass (*Megathyrsus maximus*) and signal grass (*Brachiaria* spp.) supply saponins and polyphenol compounds that modulate methanogenesis [32]. Mixed swards incorporating legumes such as white clover (*Trifolium repens*) and red clover (*Trifolium pratense*) elevate isoflavone content, particularly formononetin and biochanin A, which transfer to milk and influence fatty acid profiles [47,48]. The phytochemical density and composition of these forages are strongly influenced by plant maturity, with younger vegetative growth typically containing higher concentrations of bioactive secondary metabolites compared to mature reproductive stages [43,49].

### 3.2. Legumes, Shrubs and Tree Fodder

Sources of phytochemicals in dairy nutrition extend beyond traditional feeds to include agro-industrial byproducts and specialized plants, offering cost-effective and sustainable supplementation opportunities. For instance, tropical legumes and shrubs provide secondary metabolites like tannins and saponins that mitigate enteric methane while improving ruminant nutrient utilization [32]. In meat production, similar sources influence carcass quality by modulating lipid metabolism and oxidative stability. Linehan et al. [49] reviewed organic milk production, noting that phytochemical-rich forages in pasture-based systems elevate antioxidants and fatty acids, contributing to superior nutritional properties. Integrating these sources into ruminant diets requires consideration of regional availability, such as using willow fodder in temperate areas for tannin-based methane reduction or clove extracts in tropical systems for antimicrobial benefits [33,50].

Condensed-tannin-rich legumes (e.g., *Lotus* spp., sainfoin) and browse species (e.g., *Acacia*, *Leucaena*) are particularly relevant because they supply moderate levels of tannins and saponins that can reduce methane yield, improve nitrogen utilization and shift milk and meat fatty acid profiles towards more unsaturated and health-promoting patterns [32,51,52,53]. In low-input and semi-arid grazing systems, shrubs and tree fodders therefore represent both a resilience strategy to fodder scarcity and an important phytochemical reservoir for methane mitigation and parasite control [3,32,34].

### 3.3. Crop Residues and Agro-Industrial Byproducts of Plant Origin

Agro-industrial by-products such as grape pomace, olive cake, citrus pulp, tomato pomace and vegetable peels can also be valuable carriers of polyphenols, flavonoids and other phytochemicals in ruminant diets, while simultaneously contributing to circular bioeconomy goals [41,54,55]. Nevertheless, their phytochemical content is highly variable and influenced by processing conditions (e.g., pressing, drying, extraction), so careful characterisation and diet formulation are required to avoid excessive tannin or lignin intake and ensure consistent animal responses [3,32,41].

### 3.4. Extracts and Commercial Phytogenic Additives

Integrating these sources into ruminant diets requires consideration of regional availability. Table 1 expands on common dietary phytochemical sources, incorporating examples relevant to milk and meat production, with key bioactive properties and references from recent literature. In addition to whole plants and by-products, an increasing number of commercial phytogenic additives based on essential oils, oleoresins, tannin or saponin extracts and polyherbal blends are now used in ruminant rations [24,56,57]. These products typically standardise specific bioactive components (e.g., thymol, carvacrol, cinnamaldehyde, capsaicinoids or quebracho tannins) and are supplied as premixes, boluses, mineral blocks or encapsulated forms to improve palatability and rumen stability [56,58,59].

## 4. Mechanistic Insights

The intricate mechanisms by which phytochemicals modulate rumen microbiota and subsequently influence mammary gland metabolism represent a complex network of biochemical interactions that fundamentally alter ruminant production systems. These plant-derived bioactive compounds, including tannins, saponins, essential oils, and flavonoids, exert their effects through multiple interconnected pathways that reshape microbial ecology, fermentation dynamics, and host metabolism [3].

### 4.1. Mechanisms of Antimicrobial, Antimethanogenic and Rumen Fermentation Modulation Activities

Phytochemicals primarily interact with rumen microorganisms through direct antimicrobial mechanisms and indirect metabolic perturbations. The fundamental mode of action involves disrupting microbial cell membrane integrity, where compounds like tannins and essential oils increase membrane permeability and fluidity, leading to cellular metabolite efflux and eventual microbial death [8]. Specifically, condensed tannins demonstrate selective inhibition of Gram-positive bacteria while showing reduced efficacy against Gram-negative species due to differences in cell wall composition [9].

The mechanistic basis for methanogenic suppression involves multiple pathways. Saponins interact with sterol in protozoal membranes, causing defaunation and reducing methanogenic archaea populations that maintain symbiotic relationships with protozoa [34]. Essential oils, particularly those containing polyphenol compounds like thymol and carvacrol, disrupt the proton motive force and electron transport chains in methanogens, directly inhibiting their metabolic activity [32]. In a hydrogen-centred view of rumen fermentation, these antimethanogenic effects can be interpreted as interventions on metabolic hydrogen disposal pathways rather than simple antimicrobial actions. By decreasing the abundance or activity of methanogens and their protozoal partners, phytochemicals redirect reducing equivalents toward alternative sinks such as propionate formation, microbial biomass and, to a lesser extent, reductive acetogenesis [32,60,61]. This concept is consistent with evidence that low-methane phenotypes and methanogenesis-inhibited systems rely on enhanced propionate-producing consortia and altered hydrogenotrophic networks to maintain redox balance [60,62].

A critical mechanistic insight involves phytochemical interference with ruminal biohydrogenation processes. Tannins selectively inhibit specific bacterial populations responsible for the final saturation steps of dietary polyunsaturated fatty acids, particularly the conversion of vaccenic acid to stearic acid [52]. This occurs through tannin–protein complex formation that reduces substrate availability for biohydrogenating bacteria, along with direct antimicrobial effects on targeted microbial groups [9]. At the molecular level, these actions stem from interactions between phytochemicals and bacterial enzymes involved in fatty acid metabolism. Hydrolyzable tannins, in contrast, exert modulatory rather than strongly inhibitory effects on biohydrogenation, enabling the controlled accumulation of beneficial intermediates such as conjugated linoleic acid and trans-11 18:1 [52]. These differing responses between tannin types reflect their distinct chemical structures and binding affinities with microbial substrates [3]. Comprehensive reviews on plant polyphenols and tannins confirm that these compounds modulate not only the last steps of biohydrogenation but also upstream lipolysis and isomerization reactions, mainly by targeting *Butyrivibrio* and related taxa, thereby altering the spectrum of rumen-derived fatty acid intermediates [9,52,63]. Such changes in ruminal lipid metabolism translate into higher outflow of health-promoting intermediates to the small intestine and ultimately to milk and meat, but they are clearly dose-dependent and influenced by the structure and protein-binding capacity of the tannins [52].

Phytochemicals alter ruminal fermentation stoichiometry by redirecting metabolic hydrogen away from methanogenesis toward propionate production through the succinate and acrylate pathways and via reductive acetogenesis. This shift is driven by inhibition of hydrogen-producing bacteria and stimulation of alternative electron-using microbial groups, including propionate-producing consortia, effectively creating alternative electron sinks [60,61,62,64]. Saponins, particularly those from Quillaja and Yucca species, demonstrate aforementioned effect by selectively targeting specific bacterial populations while preserving beneficial fibrolytic species [65]. The shift toward propionate production has profound implications for host energy metabolism, as propionate is a primary gluconeogenic precursor in ruminants. This metabolic redirection enhances energy efficiency and supports increased milk production through improved glucose availability for lactose synthesis [66]. Additionally, the reduction in acetate: propionate ratios influence mammary gland lipogenesis, affecting milk fat composition and potentially improving the nutritional profile of dairy products [67]. Meta-analyses of in vivo trials with essential oils, flavonoids and saponin-rich extracts support this mechanistic framework, showing consistent, albeit modest, increases in propionate proportion, improvements in nitrogen utilisation and small reductions in methane emissions when phytochemicals are supplied within optimal dose ranges [20,21,22,68]. These quantitative syntheses underline that phytochemicals act more as fine-tuning agents of fermentation patterns than as “all-or-nothing” inhibitors, and that background diet and class of compound strongly modulate the magnitude of the response [69].

Enhanced propionate production increases gluconeogenic substrates, supporting lactose synthesis and milk yield, while concurrently, the modulation of ruminal biohydrogenation preserves beneficial fatty acid intermediates, which directly influence mammary lipid metabolism by increasing the levels of conjugated linoleic acid and omega-3 fatty acids in milk [52,70]. The molecular mechanisms involve altered substrate availability to mammary epithelial cells and modified expression of lipogenic enzymes. Phytochemical-induced changes in ruminal volatile fatty acid profiles affect the activity of acetyl-CoA carboxylase and fatty acid synthase in mammary tissue, influencing de novo fatty acid synthesis [71]. Furthermore, the increased flow of beneficial fatty acid intermediates from the rumen provides substrates for tissue desaturase enzymes, particularly Δ9-desaturase, enhancing the production of health-promoting fatty acids such as cis-9, trans-11 conjugated linoleic acid, other MUFAs like cis-9 18:1 from palmitic and stearic acids, respectively, in milk and meat [52]. Multi-omics and systems-biology approaches strengthen the concept of a rumen–mammary axis responsive to phytochemicals. Studies combining microbiome, metabolome and host phenotypes have shown that changes in key bacterial genera (e.g., *Prevotella*, *Succinivibrionaceae* and specific fibrolytic taxa) are linked to shifts in amino acid, carbohydrate and lipid metabolites that explain individual differences in milk yield, energy-corrected milk and milk fatty acid profiles [64,65,66,67]. These data suggest that phytochemicals may enhance milk production efficiency by simultaneously steering rumen fermentation toward glucogenic profiles and increasing the supply of bioactive lipid intermediates to the mammary gland [70,71].

The mechanistic effects of phytochemicals on rumen microbiota extend beyond simple antimicrobial activity to include complex community restructuring. Different phytochemical classes demonstrate selective pressure on specific microbial populations, with tannins particularly affecting Gram-positive bacteria and saponins showing preferential activity against protozoa [9]. This selectivity results from differential membrane compositions and metabolic pathways among microbial groups [8]. The restructuring process involves competitive exclusion mechanisms where phytochemical-resistant bacteria gain competitive advantages, leading to community composition shifts favoring beneficial fermentation patterns. Essential oils demonstrate this effect by inhibiting specific bacterial populations while promoting the growth of others, ultimately enhancing propionate production and reducing methane emissions [32].

Recent metabolomic studies reveal that phytochemicals create complex networks of metabolite–microbe interactions that extend beyond the rumen to influence host metabolism. Specific bacterial taxa, particularly *Prevotella* species, strongly correlate with metabolites involved in amino acid, carbohydrate, and lipid metabolism [66]. These interactions suggest that phytochemical-induced changes in microbial composition directly influence the metabolic landscape available to the host animal [65]. The mechanistic basis for these interactions involves altered production of microbial metabolites that serve as signalling molecules and metabolic precursors for host tissues.

### 4.2. Mechanisms of Antioxidant and Anti-Bloat Activities

Phytochemicals such as tannins, saponins, essential oils, and flavonoids exert antioxidant and anti-bloat activities in ruminants via interconnected redox, microbial, and fermentation pathways that are strongly structure- and dose-dependent [3]. Condensed and hydrolysable tannins provide direct antioxidant protection by scavenging reactive oxygen species, chelating transition metals, and inhibiting pro-oxidant enzymes, with antioxidant capacity closely linked to their degree of polymerization and polyphenol hydroxyl density [3,72]. Within the gastrointestinal tract, these polyphenols reduce lipid peroxidation, stabilize endogenous antioxidants, and form protein–tannin complexes that limit oxidative damage while also modulating biohydrogenation and preserving polyunsaturated fatty acids in milk and meat [3,9]. Indirect antioxidant effects arise from reshaping the rumen microbiome, where phytogenic feed additives rich in polyphenols and essential oils selectively suppress proteolytic and hyper-ammonia–producing bacteria, decrease methanogenic archaea, and favor bacterial consortia associated with improved redox balance and lower oxidative stress [8,9,34]. Anti-bloat activity is primarily mediated through suppression of gas-producing and foam-stabilizing microbial groups: saponins disrupt protozoal membranes, reducing protozoa and their associated methanogens, while tannins and essential oils further inhibit methanogenesis and hydrogen-producing microbes, thereby lowering total gas production and methane yield and shifting fermentation toward propionate with reduced acetate–propionate ratios [73,74,75]. These changes in fermentation patterns, combined with tannin-induced reductions in fiber degradation and modifications of protein precipitation and surface-active components at the liquid–gas interface, decrease rumen foam stability and bloat risk, positioning phytochemicals as mechanistically robust tools to concurrently mitigate oxidative stress and frothy bloat in sustainable ruminant production systems [3,8].

### 4.3. Mechanisms of Protein/Nutrient Protection and Growth Promoting

Phytochemicals exert sophisticated mechanisms of protein and nutrient protection that fundamentally enhance growth promotion in ruminants through multiple interconnected pathways. Condensed tannins demonstrate the primary mechanism by forming stable protein–tannin complexes that precipitate with soluble proteins, effectively reducing ruminal protein degradation and increasing dietary protein utilization [76]. This protein protection occurs through hydrogen bonds between polyphenol groups of tannins and carboxyl groups of protein chains, creating complexes that remain stable at ruminal pH (6–7) but dissociate in the acidic abomasum (pH < 3.5), thereby increasing metabolizable protein flow to the small intestine [76]. The optimal tannin concentration for protein protection ranges from 20–40 g/kg dry matter, which can increase abomasal protein flow by up to 53% and small intestine absorption by 59% without compromising apparent digestibility [76].

Plant bioactive compounds, particularly tannins and polyphenol oxidase, provide effective protection against excessive proteolysis by binding proteins and reducing soluble nitrogen content, leading to improved nitrogen use efficiency and better animal performance [77]. Moderate tannin levels (3–4% dry matter) can precipitate with soluble proteins and increase protein supply to ruminants by binding proteins and limiting ruminal breakdown, resulting in enhanced weight gain [51]. The growth-promoting effects are mediated through increased microbial protein synthesis, as tannins reduce ruminal protein digestion while simultaneously decreasing ruminal nitrogen recycling and inhibiting methanogenic populations through reduced hydrogen production [76].

Phytochemical supplementation demonstrates significant growth promotion through altered rumen fermentation patterns. Tannin and Capsicum species combinations increase milk yield (37.9 vs. 36 kg/d), energy-corrected milk (39.7 vs. 37.1 kg/d), and protein yield (1.15 vs. 1.08 kg/d) compared to control diets [67]. These improvements correlate with increased total volatile fatty acid concentrations (118.1 vs. 101.5 mM) and elevated β-hydroxybutyrate levels (0.49 vs. 0.42 mmol/L), indicating enhanced energy metabolism [67]. The mechanism involves partial manipulation of rumen microbiota, specifically reducing populations of *Selenomonas ruminantium*, *Succinimonas amylolytica*, and *Streptococcus bovis*, which are associated with low feed efficiency [67].

Essential oils and saponins contribute to nutrient protection through distinct mechanisms, inhibiting rumen ammonia production and decreasing urinary nitrogen excretion [78]. Saponins disrupt protozoan membranes, reducing both protozoa and methanogenic archaea populations, while essential oils impair archaeal energy metabolism, collectively improving nutrient utilization efficiency [32]. Secondary metabolites including tannins, saponins, flavonoids, and essential oils act as growth promoters and immune boosters, with great potential for rumen manipulation and significant roles in improving animal production and reproduction [79]. These phytochemical mechanisms collectively redirect nitrogen excretion from urine to feces, reducing environmental nitrogen losses while maintaining or improving animal growth performance, representing a sustainable approach to enhancing ruminant production efficiency [77].

## 5. Dose–Response Evidence

Understanding dose–response relationships is critical to defining safe and effective inclusion levels of phytogenic feedstuffs in ruminant diets. Across phytochemical classes, responses typically follow a hormetic-like pattern, with beneficial effects on intake, digestibility, performance, and oxidative status at low-to-moderate doses, and neutral or detrimental effects when inclusion rates exceed relatively narrow thresholds [3]. For tannins, dose–response evidence reveals species-dependent thresholds. Condensed tannins in sheep diets optimize at 2–3% DM, enhancing protein utilization and methane mitigation, but exceeding 4% DM leads to depressed intake and digestibility due to enzyme inhibition and astringency [51]. Though cattle show intermediate sensitivity, goats tolerate higher levels owing to proline-rich saliva and tannin-resistant microbes. Hydrolyzable tannins, metabolized differently, modulate biohydrogenation at moderate doses but risk hepatotoxicity above 5% DM, as seen with pomegranate peel, elevating serum alanine aminotransferase (an enzyme mainly found in the liver) [9,80].

Phytochemicals enhance protein protection and growth promotion through documented mechanisms that overcome baseline variability challenges in ruminant systems. Tannin-containing phytogenic blends demonstrate consistent protein-sparing effects, increasing milk yield and protein yield despite breed variations [67]. Essential oils modulate rumen microbiota composition, with mangosteen peel liquid reducing methanogens by 30.6% while improving microbial nitrogen synthesis efficiency by 15.8% [81]. Phytogenic compounds consistently enhance milk nitrogen efficiency and feed conversion regardless of baseline parameters [82]. Growth promotion mechanisms involve increased butyric acid concentrations and improved volatile fatty acid profiles, with phytogenic blends showing superior antioxidant responses across different management systems [83]. Despite dosing standardization challenges, phytochemicals demonstrate 5–13% improvements in body weight gain across species through antimicrobial and immunomodulatory pathways [84]. The underlying mechanisms involve mucosal antibody production and inflammatory cytokine suppression, providing consistent benefits despite baseline variability [85]. However, only 20 of over 500 tested compounds have progressed to in vivo validation, highlighting standardization needs [86]. Polyphenol compounds reduce ruminal proteolysis while saponins and essential oils redirect metabolic hydrogen toward beneficial volatile fatty acids [3,8]. Interactive effects between protein levels and phytogenic supplementation consistently improve nutrient digestibilities and rumen fermentation efficiency [87]. Table 2 summarizes key dose–response patterns across phytochemical classes, highlighting optimal ranges and thresholds for avoiding adverse outcomes. Future research should standardize protocols across classes and species for sustainable application. Furthermore, dose–response trials should incorporate omics technologies to refine thresholds, ensuring balanced milk and meat systems enhancements while advancing sustainability goals.

## 6. Impact of Dietary Phytochemicals on Production of Ruminants

### 6.1. Effects on Feed Intake

Phytogenic feedstuffs are promising phytochemical sources that can replace traditional growth promoters in livestock diets, enhancing productivity and product quality. However, their effects, particularly in ruminants, remain inconclusive, necessitating further in vivo studies to optimize their application and elucidate mechanisms of action [69]. Flavonoids and saponins enhance feed palatability and ruminant intake while offering benefits like reduced methane emissions and improved gut health. Balancing the inclusion rates of phytogenic forages, crude extracts, and purified bioactive compounds in ruminant diets is essential to prevent potential anti-nutritional or toxic effects and to optimize nutrient utilization and overall animal performance.

### 6.2. Effects on Key Performances

#### 6.2.1. Milk and Meat Yield

##### Milk Yield

Milk production in ruminants requires nutritional strategies that support high metabolic demands for energy, amino acids, and rumen fermentation efficiency. Phytogenic feed additives, including tannins, saponins, essential oils, capsaicinoids, and polyherbal formulations, have demonstrated potential to improve milk yield primarily through enhanced nitrogen utilization, improved rumen fermentation, and increased feed efficiency.

Tannins play a key role in dairy systems by improving nitrogen utilization and supplying amino acids required for milk synthesis. Both condensed and hydrolyzable tannins enhance milk yield by increasing bypass protein availability while mitigating bloat and intestinal parasitism in ruminants [88]. Appropriate inclusion levels are critical to avoid adverse effects on rumen microbial activity and to maintain animal health, thereby ensuring sustainable milk production. Supplementation of tannins combined with Capsicum has been shown to enhance milk yield and energy-corrected milk production in dairy cows by improving rumen fermentation efficiency, increasing volatile fatty acid concentrations, and enhancing the utilization of rumen undegradable protein [67]. Saponins also contribute to improved milk production by enhancing energy utilization and feed efficiency. Through rumen defaunation and reduced protozoa-mediated protein degradation, saponins improve nitrogen utilization, resulting in increased milk output [14]. Essential oils and capsaicin-based phytogenics further support lactation performance. Capsaicin supplementation increased milk yield by 2.9% and improved feed efficiency by 3.4% in dairy cows, particularly under stress conditions, highlighting its role in supporting lactation during environmental or physiological challenges [37]. Across dairy systems, most essential-oil- and capsaicin-based additives induce modest increases in milk yield (often <5%) or improve feed efficiency, with neutral responses frequently observed when basal diets are already nutritionally optimized [3,8,20,26,37,89].

Polyherbal phytogenic additives exhibit dose-dependent effects on milk production. In lactating Damascus goats, dietary inclusion of mustard and cumin seeds significantly enhanced milk yield, with cumin supplementation increasing actual milk yield by 11.1% and energy-corrected milk yield by 15.4%, compared with a 6.8% improvement observed with mustard supplementation [36]. In dairy ewes, supplementation with a standardized mixture of bioactive compounds derived from thyme, anise, and olive, particularly in rumen-protected form, resulted in a substantial increase in milk yield without adversely impacting other milk properties. The most pronounced responses were observed between the seventh and ninth weeks of supplementation [3].

Dietary background markedly influences the response to phytogenic additives. In high-forage or low-quality forage-based systems, particularly in tropical or low-input dairy production, herbal mixtures rich in essential oils and polyphenols improve nutrient digestibility, milk yield, and milk protein content, indicating a greater responsiveness under nutritionally limiting conditions [90]. Conversely, in high-concentrate dairy diets, phytogenics more commonly improve feed efficiency or milk composition rather than total milk yield [34,35].

##### Meat Yield

Meat production systems differ fundamentally from dairy systems in nutritional objectives, metabolic priorities, and feeding strategies, with emphasis placed on growth rate, feed efficiency, muscle accretion, and carcass characteristics. Consequently, the effects of phytogenic feed additives on meat yield are more variable and strongly influenced by phytogenic type, inclusion rate, and diet composition.

Tannins affect meat yield primarily by modifying protein digestion patterns. Condensed tannins can increase the flow of bypass protein to the small intestine, potentially enhancing amino acid availability for muscle growth; however, effects on final meat yield remain inconsistent across studies and depend on tannin concentration and dietary context [3]. Excessive tannin levels may impair rumen microbial activity, highlighting the importance of appropriate dosing. Saponins improve meat production by enhancing energy utilization and feed efficiency. Through rumen defaunation and reduced protozoa-driven protein turnover, saponins improve nitrogen utilization, supporting muscle accretion and growth performance [14]. Saponin-rich plant extracts, such as tea seed saponins administered at 13.8–32.2 mg/kg body weight in cattle, have been associated with improved growth performance and increased meat yield potential, although effects on final carcass traits require further validation [3].

Essential oils generally exert positive effects on meat yield, though responses are inconsistent. Crossbred F1 Angus × Nellore bulls supplemented with a blended phytogenic additive containing licorice, caraway, cinnamon, and vanilla at 150 ppm exhibited a 15 kg increase in carcass weight compared with controls, without changes in dressing percentage, indicating improved meat yield efficiency [44]. Polyherbal additives further demonstrate dose-dependent effects; BioCholine^®^ supplementation at 7.5 g/kg DM in finishing lambs linearly increased longissimus dorsi muscle area, suggesting enhanced muscle development and lean meat production potential [68].

Diet composition strongly modulates phytogenic efficacy in meat production. In high-concentrate finishing diets, phytogenic additives primarily enhance feed conversion efficiency and carcass weight through improved nutrient utilization and rumen fermentation modulation [8,78]. In contrast, under high-forage or medium- to low-quality forage diets, phytogenics rich in polyphenols and essential oils improve nutrient digestibility and growth efficiency, indirectly supporting increased meat yield [90]. Additionally, polyphenol compounds provide antioxidant benefits that reduce oxidative stress, support growth performance, and improve meat quality and shelf life [91,92].

Despite the demonstrated potential of phytogenic feed additives to improve milk and meat yields, responses remain inconsistent in ruminants compared with monogastric animals [69]. This variability reflects differences in production goals, dietary composition (high-forage versus high-concentrate systems), animal physiological status, and phytogenic formulation. These findings underscore the need for standardized supplementation strategies and diet-specific optimization of phytogenic inclusion levels to achieve consistent productivity gains while maintaining animal health and welfare standards across diverse ruminant production systems [57,93].

#### 6.2.2. Milk and Meat Composition

Phytochemical interventions have demonstrated significant potential to modify milk and meat composition in livestock, enhancing nutritional value and functional properties through various bioactive mechanisms. Fatty acid composition represents one of the most extensively studied aspects of phytochemical effects on milk and meat. Tannin supplementation has shown a remarkable ability to modulate ruminal biohydrogenation, consequently altering fatty acid profiles in meat and milk [52]. Condensed tannins effectively increase concentrations of potentially beneficial fatty acids, including 18:3n-3, 18:2n-6, trans-11 18:1, and CLAs in meat and milk [63]. These improvements contribute to meeting consumer demand for health-promoting foods with enhanced omega-3 fatty acid content.

**Figure 1:**
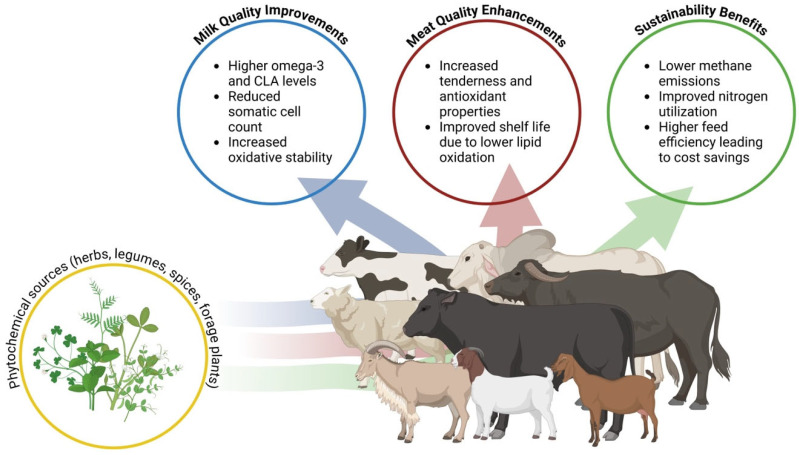
Effects of dietary phytochemicals on ruminant production, quality, and sustainability.

In milk, savory plant inclusion enhanced fatty acid composition, increasing CLAs and n-3 fatty acids while reducing saturated fats, with fish oil further amplifying these improvements in healthy fatty acids [34]. Providing cumin seeds to lactating Damascus goats positively influenced milk composition by increasing fat and lactose content and improving the milk fatty acid profile, lowering milk saturated fatty acids (SFA) by 3.9% and increasing total unsaturated fatty acids (UFA) by 9.7% and CLAs by 23.1%, while mustard seeds contributed to beneficial changes but had less pronounced effects than cumin [36]. The higher ruminal short-chain fatty acid (SCFA) production with cumin compared to mustard may be attributed to differences in their phytoconstituents, although the specific biological activity of these compounds remains unknown. Capsaicin improved milk fat concentration and yield by 2.6% and 4.0%, respectively, likely due to enhanced rumen fermentation and increased precursors like butyrate and β-hydroxybutyrate [37]. Although milk protein concentration showed a slight decrease, likely due to a dilution effect associated with increased milk volume, the overall protein yield remained unchanged. This indicates that capsaicin may modulate metabolic activity and rumen microbial function in a manner that supports sustained protein synthesis while contributing to improvements in overall milk quality. Flavonoids increase unsaturated fatty acid concentrations in milk, while tannins enhance milk protein content by protecting dietary protein from ruminal degradation. Including tannins and Capsicum in dairy cow diets improves milk protein synthesis and overall milk composition by partially modulating rumen microbiota, reducing bacteria associated with low feed efficiency, and optimizing nutrient availability for milk production [67]. As shown in Figure 1, phytochemical sources like herbs and legumes contribute to milk quality improvements such as higher omega-3 and CLA levels, reduced somatic cell count, and increased oxidative stability.

Polyherbal phytogenic additives and specific phytochemical compounds have demonstrated targeted effects on ruminant body composition. Supplementation with BioCholine^®^ at various doses (2.5, 5.0, and 7.5 g/kg DM) in finishing lambs did not affect cooking loss, protein, fat, moisture, and collagen content of meat [68], suggesting that these additives can improve growth performance without compromising fundamental meat composition characteristics. Hemp by-products show potential for enhancing ruminant meat production and preservation through their bioactive compounds [54]. Similarly, herbal plant incorporation in ruminant diets affects fat content and distribution, water content, and collagen content [94]. In a crossover trial (two groups of 4 cows) that received AFB1-contaminated feed (5 ± 1 µg/kg) for 10 days, supplementation with turmeric powder (20 g/head/day) was associated with a tendency for lower average AFM1 concentrations in milk during the last four days of treatment, but the reduction was not statistically significant [28]. Milk yield and compositional parameters remained unchanged with turmeric supplementation, suggesting that the conventional turmeric powder used in the study may have been insufficiently bioavailable to produce measurable physiological effects. As shown in Table 3, dietary phytochemicals improve both milk and meat productivity and quality by enhancing yields and enriching products in beneficial fatty acids and bioactive compounds.

These compositional changes’ mechanisms involve complex interactions between phytochemicals and rumen microbiota, affecting nutrient metabolism and deposition patterns [8,15]. Phytochemicals can modulate rumen fermentation to mediate fermentation kinetics through diet–microbe interactions, ultimately influencing meat composition [8]. Recent advances in understanding phytochemical impacts suggest potential epigenetic effects on ruminant health and production, which may have long-lasting implications for meat composition and quality [47]. However, the effectiveness of phytochemicals in modifying milk and meat composition varies considerably among studies, highlighting the need for standardized approaches and a better understanding of optimal application strategies.

## 7. Influence of Diet and Production on Phytogenic Additive Efficacy in Ruminants

Ruminant responses to phytogenic additives are often weaker and more variable than those seen in monogastric species. For example, recent systematic reviews report that lambs can show up to ~30% growth improvements with herbal supplements, whereas cattle gains are consistent but modest, implying phytogenics are more complementary than primary promoters in cows [84]. In ruminants, factors like production stage and health status further confound outcomes. Phytogenics generally give the biggest boosts under challenge or high-demand conditions (e.g., disease stress, early lactation), rather than uniformly across all herds.

### 7.1. Diet Composition and Feeding System

The type of diet and production regimen strongly modulate PFA effects. In high-energy, concentrate-rich rations (feedlot or TMR systems), phytogenic blends have shown reliable benefits. Rivera-Chacón et al. [103] found that adding menthol, thymol, and eugenol to an acidogenic (high-grain) diet raised rumen pH and increased acetate-to-propionate ratio, thereby stabilizing fermentation and reducing the risk of subacute acidosis. In a feedlot study, cattle fed a phytogenic blend (tannins, flavonoids, essential oils) on ~90% grain diets ate slightly less but maintained growth, yielding about a 3.3% numerical improvement in feed efficiency [104]. Likewise, in confined dairy cows fed a total mixed ration, phyto-additives (alone or combined with yeast/minerals) improved energy-corrected milk yield, milk fat percentage, and feed efficiency compared to cows fed monensin [105]. These effects suggest that under intensive feeding (high concentrate TMR or finishing rations), phytogenics can consistently optimize rumen fermentation and energy use. In contrast, responses are often muted in forage-heavy or variable grazing systems. For instance, in dairy goats on mixed diets, multi-herb supplements did not alter milk volume but significantly increased milk fat percentage [106], indicating stable yields with improved composition.

### 7.2. Standardization and Optimization of Supplementation

Because PFA responses depend so much on context, tailored strategies are essential. Studies emphasize non-linear dose effects and the need for precise dosing [84]. Optimal inclusion levels likely differ by diet type and production goal: intensive, high-forage rations may require lower doses, whereas high-grain diets might need specific compound blends to avoid negative impacts. For example, combining phytogenics with probiotics or minerals has produced synergistic gains in milk fat yield and lowered inflammation markers in cows [105]. Overall, ensuring consistent productivity gains with PFAs will require customizing formulations and doses to each feeding system and animal group. Tailored supplementation, considering forage-to-concentrate ratio, physiological state, and stress factors, can transform the otherwise variable phytogenic response into a dependable tool for enhancing ruminant performance [84,103].

## 8. Impact on Animal Health and Sustainability

### 8.1. Greenhouse Gas (GHG) Emission Reduction

Ruminant livestock are major contributors to methane and ammonia emissions, leading to substantial energy and protein losses that lower feed efficiency and negatively affect the environment. Phytochemicals suppress methane production by modifying rumen fermentation patterns, typically increasing propionate and total volatile fatty acids (VFAs) while decreasing the acetate-to-propionate ratio and ammonia nitrogen (NH_3_-N) levels [41]. Their antimethanogenic and antiprotozoal activities collectively enhance rumen efficiency. The efficacy of phytochemicals varies depending on their source, chemical structure, and dietary inclusion level. Phytochemicals in clove (*Syzygium aromaticum*), particularly eugenol, have been shown to reduce ruminal methane and ammonia production by selectively inhibiting methanogenic archaea and deaminating bacteria [50]. These microbial shifts alter the rumen fermentation pattern, increasing the proportions of propionate and butyrate while enhancing overall protein utilisation. Consequently, the incorporation of clove extracts as feed additives can improve feed efficiency, support higher milk yield, and contribute to more sustainable livestock production systems.

**Figure 2:**
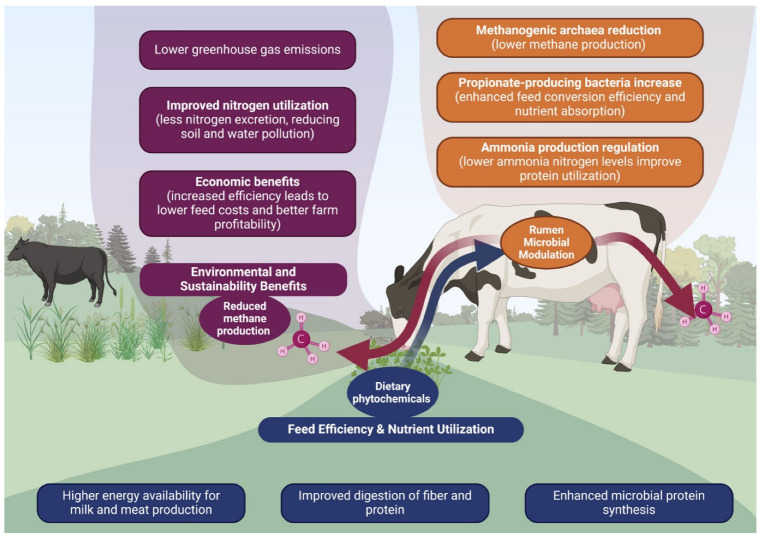
Role of Phytochemicals in Reducing Methane Emissions and Enhancing Feed Efficiency.

Other phytochemical classes, including tannins, saponins, and essential oils, also offer eco-friendly alternatives to antibiotic-based rumen modifiers. Tannins can decrease methanogenesis and enhance feed efficiency through their inhibitory effects on rumen methanogens and protozoa [34]. Saponins improve fermentation characteristics, nutrient digestibility, and microbial protein synthesis while reducing methane emissions [59]. Essential oils modulate rumen microbial communities, contributing to improved nutrient utilisation and lower methane output [34,107]. As illustrated in Figure 2, these phytochemicals collectively act through mechanisms such as reducing methanogenic archaea, increasing propionate-producing bacteria, and regulating ammonia formation. Together, they present a promising strategy for improving ruminant health, productivity, and environmental sustainability, although further research is needed to refine optimal dosages and evaluate cost-effectiveness.

According to Singh et al. [17], saponins and tannins suppress methanogenic archaea, lowering methane output while improving energy retention. Phytochemicals, including flavonoids, terpenoids, and alkaloids, offer promising benefits for sustainable ruminant production by enhancing nutrition, reducing methane emissions, and supporting disease prevention and treatment [3]. However, their mechanisms and applications require further research to optimize their use as alternatives to antibiotics and anthelmintics in ruminant diets. While traditionally plant secondary metabolites were considered as anti-nutritional, recent studies highlight their potential to manipulate rumen fermentation favourably and contribute to safer and more sustainable food production systems by reducing the environmental impact of ruminant agriculture [108].

Research has shown that interventions like tannins and essential oils can reduce methane yield in cattle and sheep, depending on diet and dose, without compromising digestibility [56]. For example, although macroalgae and 3-nitrooxypropanol demonstrate high methane-reduction efficacy, plant phytochemicals provide a more sustainable and widely applicable alternative for global livestock systems [5]. In meat production, lowered methane correlates with improved energy partitioning toward muscle growth, enhancing carcass quality and feed conversion ratios. Cobellis et al. [56] reviewed how anti-methanogenic compounds, including polyphenols, alter ruminal microbiota to favor propionate over acetate, reducing hydrogen availability and benefiting milk fat synthesis and meat marbling. Phytogenic additives from trees and shrubs, such as those containing saponins, achieve methane cuts by modulating protozoal populations, with additive effects on health by reducing bloat and parasites [34]. Sustainability is amplified in low-input systems, where forages rich in secondary metabolites naturally curb emissions, as seen in tropical legumes that integrate well with mixed farming [32]. However, response variability underscores the need for tailored strategies to avoid production losses.

Phytochemicals in willow fodder blocks, rich in condensed tannins, can reduce methane emissions in sheep by up to 20% per unit of metabolic body weight, highlighting their potential as a natural strategy for mitigating greenhouse gas emissions in ruminant production systems [33]. Phytochemicals such as condensed tannins, saponins, essential oils, and flavonoids effectively modulate the rumen microbiome, reducing methane emissions in ruminants by up to 26% [32]. Incorporating tropical legumes and plants rich in these secondary metabolites into ruminant diets offers a sustainable approach to mitigating greenhouse gas emissions while improving nutrient utilization. Phytogenic feed additives, including savory, reduce methane emissions by altering rumen microbial ecology, lowering ruminal ammonia concentration, and improving VFA profiles [107]. As illustrated in Figure 2, dietary phytochemicals lead to rumen microbial modulation. This visual emphasizes how phytochemical-driven changes in rumen dynamics foster health resilience and ecological balance in dairy and beef systems. These natural compounds offer an eco-friendly alternative to synthetic additives, promoting sustainability and addressing climate change concerns. Nevertheless, optimizing their use requires balancing methane reduction with impacts on animal growth and production efficiency. Beyond absolute methane yield, dietary phytochemicals also influence methane emission intensity, expressed per unit of energy-corrected milk or carcass weight, which is a more relevant metric for climate policy and farm-level sustainability assessment. Integrative evaluations indicate that moderate inclusion levels of tannins, saponins and essential oils can reduce emission intensity while maintaining, or even slightly improving, productive performance when diets are carefully formulated [5,19,32]. Recent modelling and scenario analyses further suggest that phytogenic strategies must be combined with other mitigation levers, such as genetic selection, improved forage quality and manure management, to approach sectoral greenhouse gas reduction targets [19,48,109]. Within this portfolio, phytochemicals provide a scalable option for both intensive and pasture-based systems, especially where locally available shrubs, legumes and tree fodders naturally supply secondary metabolites [32,33,108].

### 8.2. Nitrogen Utilization

Improved nitrogen efficiency reduces nitrogen excretion, minimizing environmental pollution. Phytochemicals enhance nitrogen utilization in ruminants by modulating rumen degradation and microbial synthesis, which are crucial for sustainable milk and meat production. Extracts from plants rich in polyphenols improve nitrogen retention in sheep, reducing urinary losses and supporting microbial protein yield [78]. In dairy cows, mixtures of phytochemicals such as those from essential oils increase rumen undegradable protein, boosting milk nitrogen efficiency and lowering manure nitrogen output [67]. For meat animals, tannins from quebracho protect dietary protein from excessive breakdown, enhancing post-ruminal absorption and lean tissue accretion while reducing ammonia emissions [88]. Natural phytochemicals, such as saponins and flavonoids, reduce nitrogen excretion by optimizing urea recycling and reducing antinutrients, offering eco-friendly alternatives in high-concentrate diets [78]. Allicin from garlic inhibits urease, improving urea-nitrogen conversion to microbial nitrogen, with benefits for health by reducing ruminal acidosis risks [50]. In mixed crop-livestock systems, phytochemical-rich forages promote nutrient recycling, overall farm nitrogen losses, and support biodiversity [53]. These mechanisms align with sustainability goals, but require dose optimization to prevent anti-nutritional effects. In addition to lowering total nitrogen excretion, phytochemicals can improve nitrogen-use efficiency by shifting the partitioning of dietary nitrogen from urine toward milk or muscle. Indicators such as milk nitrogen efficiency and whole-farm nitrogen balance are positively affected when tannins, saponins and essential oils reduce ruminal proteolysis and favour microbial protein synthesis, thereby decreasing the fraction of nitrogen lost as ammonia and nitrous oxide from excreta [53,67,88,110]. Zhao et al. [78] highlighted that natural phytochemicals can complement dietary protein optimization and manure management to mitigate excreta-derived N_2_O emissions, particularly in high-input systems. Future in vivo studies should therefore systematically report nitrogen-use efficiency metrics alongside production responses to better quantify the contribution of phytochemical-based strategies to national nitrogen mitigation targets.

### 8.3. Antioxidant and Anti-Inflammatory Properties

Phytochemicals, particularly flavonoids, are crucial in neutralizing free radicals, thereby reducing oxidative stress and inflammation. Oxidative stress, characterized by an imbalance favouring oxidants over antioxidants, is a significant contributor to chronic diseases, including cardiovascular conditions, neurodegenerative disorders, and cancer [111]. Flavonoids act as potent antioxidants by stabilizing free radicals, preventing cellular oxidative damage, and mitigating inflammation-induced tissue degradation [112]. The ability of flavonoids to counteract oxidative stress has been extensively explored in human health research, with strong evidence supporting their role in immune modulation and disease prevention [113].

Beyond their general antioxidant function, flavonoids exhibit powerful anti-inflammatory properties by regulating molecular pathways involved in immune responses. Chronic inflammation, often associated with obesity, diabetes, and cardiovascular diseases, arises due to prolonged activation of inflammatory mediators [114]. Flavonoids such as quercetin and rutin suppress pro-inflammatory cytokines while enhancing the production of anti-inflammatory markers, thereby alleviating pathological inflammation [115]. This dual function of flavonoids in reducing oxidative stress and inflammation directly contributes to improved immune function by preserving cellular integrity [111].

Furthermore, in the context of mammary gland health, flavonoids such as rutin have been shown to mitigate metabolic stress during lactation by reducing oxidative markers like malonaldehyde and hydrogen peroxide while boosting the expression of antioxidant enzymes such as superoxide dismutase and glutathione peroxidase [115]. These findings suggest flavonoid-rich diets enhance mammary gland resilience and contribute to overall systemic health, making them valuable components in functional food formulations. Therefore, the protective effects of flavonoids extend beyond simple antioxidant activity, encompassing immune regulation, inflammation control, and cellular protection, thereby improving overall resilience to diseases. In ruminant production systems, comparable antioxidant and anti-inflammatory effects have been documented in vivo. During the transition period, rutin supplementation in ewes reduced mammary oxidative damage and inflammatory markers while enhancing endogenous antioxidant enzymes, indicating improved tissue resilience in early lactation [115]. In growing goat kids, dietary polyphenols improved muscle oxidative stability and colour without impairing growth performance, demonstrating that phytochemical-rich diets can enhance meat shelf life while preserving productive efficiency [116]. Taken together, these findings suggest that the antioxidant and immunomodulatory actions of flavonoids observed in human studies translate into tangible benefits for animal health and product stability in dairy and meat systems [111,115,116]. Their widespread presence in plant-based foods underscores the significance of dietary flavonoids in preventing oxidative stress-related disorders and promoting long-term health [111,112,113].

## 9. Impact of Dietary Phytochemicals on Milk and Meat Quality

### 9.1. Effects on Physical Quality

Dietary phytochemicals therefore influence not only productive performance but also a broad spectrum of physicochemical, nutritional and sensory attributes of milk and meat that determine product value, shelf life and their potential positioning as functional foods [2,10,90]. Understanding these quality-related responses is essential to designing feeding strategies that simultaneously address farm profitability, environmental sustainability and human health.

#### 9.1.1. Ultimate pH

##### Effects in Ruminant Meat Production

Ultimate pH is pivotal in determining meat quality in livestock production, influencing factors such as tenderness, color stability, shelf life, protein functionality, enzymatic reactions, and microbial stability. Dietary phytochemicals, including polyphenols and flavonoids, have been extensively studied for their potential to modulate this parameter through interactions with metabolic and microbial processes. In meat from ruminants, these compounds generally exert minimal influence on ultimate pH despite enhancing other quality aspects like antioxidant capacity.

In reality, chemical supplementation does not significantly alter ultimate pH. For example, Cimmino et al. [116] found no differences in ultimate pH between weaned goat kids fed polyphenols from olive mill wastewater at 3.2 mg/day for 78 days and control groups, although the treatment improved antioxidant status by reducing malondialdehyde levels. Similarly, a meta-analysis of 36 studies on beef and dairy cattle supplemented with flavonoids at doses ranging from 12 to 3104 mg/kg dry matter for 24 to 168 days reported no effect on meat pH, even as tenderness improved (evidenced by lower shear force) and yellowness decreased [68]. Priolo et al. [109] noted in a review comparing pasture versus concentrate feeding in cattle and sheep that grass-derived phytochemicals may increase pH variability without resulting in abnormal values, potentially affecting color (leading to darker meat with lower L*) and flavor, but not the ultimate pH itself. Further supporting this trend, Orzuna-Orzuna et al. [68] reported no measurable changes in meat pH in male Pelibuey lambs receiving a polyherbal additive at 2.5 to 7.5 g/kg dry matter for 56 days, with no accompanying effects on color or cooking loss. These findings indicate that while dietary phytochemicals enhance certain specific quality parameters, such as antioxidant capacity, they have a limited impact on ultimate pH across ruminant species, underscoring the need for additional research to clarify the underlying mechanisms.

##### Effects in Dairy Production

Phytochemicals, particularly polyphenols like flavonoids and tannins, play a crucial role in stabilizing milk pH, essential for maintaining protein functionality, enzymatic reactions, microbial stability, and overall dairy product quality. As a diverse class of polyphenols, tannins interact with milk proteins to modulate enzymatic processes, thereby preventing excessive acidification or alkalization [117]. Their antimicrobial properties further aid pH stability by regulating microbial populations involved in fermentation, as highlighted by Wróblewska et al. [118]. Specific flavonoids, such as epigallocatechin-3-gallate and chlorogenic acid, exhibit strong binding affinities for milk proteins, including casein, α-lactalbumin, and β-lactoglobulin, influencing protein aggregation, buffering metabolic fluctuations, and maintaining a balanced pH during fermentation; this is critical for curd formation and dairy protein digestion [119]. However, the stability and efficacy of polyphenols in phytogenic feed additives are highly pH-dependent, meaning that without appropriate formulation they may undergo degradation during feed processing, rumen fermentation, or storage. Therefore, advanced delivery strategies such as microencapsulation are often required to protect these compounds, control their release, and preserve bioactivity until they reach their site of action [120].

#### 9.1.2. Water Holding Capacity (WHC)

##### Effects in Ruminant Meat Production

Dietary phytochemicals exhibit variable effects on the WHC of meat, influencing key physical quality attributes such as juiciness, drip loss, syneresis, and overall product yield and stability. These effects depend on factors like compound type, concentration, species, and delivery method, with polyphenol compounds and flavonoids showing promise in modulating protein–water interactions to enhance moisture retention and structural integrity in products. In ruminant meat, for instance, species-specific responses are evident, as citrus flavonoids supplemented in lamb diets significantly reduced WHC, potentially resulting in drier meat and decreased carcass redness when measured by the press method, whereas polyphenol compounds from grape pomace improved WHC in suckling lambs, suggesting enhanced moisture retention that could benefit tenderness and palatability, though without specific numerical or statistical details provided [42,121]. Similarly, in cattle, a meta-analysis of flavonoid supplementation from various plant sources revealed no significant alteration in WHC, indirectly assessed through pH and cooking loss metrics, despite positive impacts on other traits like reduced yellowness [68]. These findings underscore the need for targeted phytochemical applications to optimize physical quality in ruminant production systems, where lambs exhibit more pronounced WHC changes than cattle.

##### Effects in Dairy Production

Paralleling these effects in dairy systems, polyphenol compounds such as tannic and gallic acids improve milk’s water-binding capacity by interacting with proteins, thereby reducing syneresis and enhancing the yield of processed dairy products through mechanisms involving hydrogen bonding and hydrophobic effects that modify protein gelation properties and structural stability. Harbourne et al. [122] showed that adding tannic acid (0.1–1% *w*/*w*) or gallic acid (0.3–1% *w*/*w*) to skim milk before acidification (with glucono-δ-lactone) accelerates gelation and increases the storage modulus (G′) of the resulting acid-milk gel. Up to 0.8% phenolic acid strengthened the protein network, reduced water mobility, and did not significantly increase syneresis; but at 1% gallic acid, G′ dropped and syneresis increased, indicating disruption of the milk gel structure [122].

Further, polyphenol–protein complexes involving whey and casein proteins in milk improve structural integrity and bioavailability, offering valuable applications in dairy stabilization and functional food development. In fermented dairy products, these interactions can reduce allergenicity while enhancing probiotic viability [118,123]. Optimizing polyphenol incorporation in meat and dairy processing, through refined delivery mechanisms and concentration thresholds, can enhance product texture, yield, and stability. Further research is needed to maximize these benefits while preserving desirable sensory attributes across species and production contexts.

### 9.2. Effects on Nutritional Composition

#### 9.2.1. Proximate Composition

##### Effects in Dairy Production

Dietary phytochemicals regulate the proximate composition of milk and meat primarily by modulating rumen fermentation kinetics, nitrogen metabolism, and feed efficiency, rather than by directly altering tissue deposition thresholds. In lactating ruminants, the inclusion of plant secondary metabolites, specifically essential oils, tannins, and saponins has been shown to stabilize or marginally improve macronutrient yields (protein, fat, and lactose) by optimizing the supply of precursors to the mammary gland. For instance, Silva et al. [101] demonstrated that supplementing dairy cows with a blend of essential oils improved feed efficiency and milk yield without compromising the percentages of protein, lactose, or total solids, suggesting that phytochemicals can enhance nutrient utilization while maintaining compositional homeostasis. Similarly, Menci et al. [124] reported that tannin supplementation in dairy cows reduced milk urea nitrogen by 10% during dry seasons, indicating improved nitrogen efficiency and protein sparing effects that prevent dietary nitrogen loss.

The specific manipulation of rumen microbiota by saponins further influences milk protein synthesis. By suppressing protozoal populations (defaunation), saponins reduce the predation of bacteria, thereby increasing the flow of microbial protein to the duodenum; this mechanism supports sustained milk protein output even when dietary protein is restricted [32]. Furthermore, recent investigations into transition cows indicate that phytochemical mixtures can promote propionate production, a key glucogenic precursor which subsequently drives lactose synthesis and osmotic milk volume [102].

##### Effects in Ruminant Meat Production

In contrast to milk, the proximate composition of meat (moisture, crude protein, intramuscular fat, and ash) appears remarkably resistant to phytochemical manipulation, exhibiting strong homeostatic regulation. Extensive trials in small ruminants have consistently shown that while phytochemicals improve oxidative stability or sensory attributes, they rarely alter the fundamental chemical breakdown of the muscle. Ortiz-Heredia et al. [125] found that finishing lambs supplemented with essential oils and calcium malate showed improved meat tenderness and pH but no significant differences in moisture, protein, or ash percentages compared to control groups. This lack of variation is corroborated by Giller et al. [126], who observed that neither maternal nor direct dietary supplementation with polyphenol-rich grape seed extract affected the dressing percentage or proximate composition (fat, protein, ash) of meat in sheep and goats. Similarly, studies on tannin inclusion in sheep diets confirmed that while parameters like drip loss may decrease, the core nutritional density which represented by protein and ash content, remains unaffected [127]. Thus, the value of phytochemicals in meat production lies in enhancing feed conversion efficiency and preservation quality rather than modifying the gross proximate profile.

#### 9.2.2. Vitamins and Minerals

##### Effects in Ruminant Meat Production

In ruminant meat, polyphenols from pasture or supplements notably increase vitamin E (α-tocopherol) content, contributing to better antioxidant capacity. For instance, grass-finished cattle on polyphenol-rich diets exhibited a 3.1-fold increase in alpha-tocopherol compared to grain-finished counterparts, and supplementation with polyphenols or proanthocyanidins in lambs and cattle raised alpha-tocopherol levels to 3.5–5.4 mg/kg muscle, often plateauing at approximately 5 mg/kg when combined with vitamin E for optimal bioactivity [128,129,130]. In meat, polyphenol-rich diets in grass-finished cattle led to significantly elevated iron and zinc concentrations, although grapeseed extract supplementation showed no additional effect on these minerals [131]. Notably, potential trade-offs exist, as certain phytochemicals may adversely affect specific nutrients; for example, tannin-containing diets in lambs reduced vitamin B12 biosynthesis without altering iron absorption, highlighting the importance of balanced supplementation to avoid compromising overall nutrient modulation [132].

##### Effects in Dairy Production

While in milk, flavonoids similarly elevate concentrations of fat-soluble vitamins like A, D, and E by influencing lipid metabolism and ensuring greater stability and bioavailability [133]. Paralleling these benefits in dairy systems, flavonoids’ antioxidant and anti-inflammatory properties reduce oxidative stress, preserving vitamin integrity during milk processing and storage, which supports enhanced nutritional value [134]. Regarding mineral profiles, phytochemicals facilitate improved bioavailability and retention of essential minerals in meat and milk, with specific compounds modulating absorption pathways and countering antinutritional factors. Similarly, in milk, organosulfur compounds enhance mineral absorption by modulating gut microbiota and mitigating the impact of antinutrients, thereby promoting higher calcium and magnesium retention crucial for bone health and metabolic functions; these compounds maintain mineral solubility and transport efficiency, contributing to an overall superior mineral profile [135,136].

Overall, most studies report moderate but consistent improvements in antioxidant vitamins, carotenoids and selected minerals in milk and meat from animals receiving phytochemical-rich diets or grazing diverse pastures [90,110,128,129,130,133]. When these compositional shifts are interpreted together with evidence on oxidative stability and sensory properties [68,109], it becomes clear that relatively small increases in micronutrient density can yield meaningful gains in product functionality, shelf life and perceived quality. Future trials should therefore integrate detailed nutrient profiling with measurements of oxidative markers and consumer-relevant traits, enabling a more precise valuation of phytochemical-based feeding strategies as tools for producing high-value, health-oriented dairy and meat products.

## 10. Sensory Attributes

Milk and meat from ruminants fed phytochemical-rich diets exhibit enhanced sensory and nutritional qualities that could meet growing consumer expectations for premium and health-oriented animal products. Feeding practices play a decisive role in shaping product composition, as phytochemical-enriched diets improve the balance of bioactive compounds such as polyphenols, carotenoids, phytosterols, and essential fatty acids [7,49]. These bioactives modulate lipid metabolism and oxidative stability, leading to a lower omega-6 to omega-3 ratio and superior sensory attributes, including creaminess, mouthfeel, and tenderness. Their antioxidant and anti-inflammatory actions also preserve protein integrity, improving milk and meat’s texture and overall structural stability.

In dairy systems, interactions between polyphenols and milk proteins enhance gel formation, reduce syneresis, and strengthen the network structure of dairy matrices. Such effects translate into better consistency and textural uniformity in fermented products like yogurt [137]. Comparable mechanisms occur in meat, where dietary phytochemicals reduce post-mortem proteolysis variability through their antioxidative activity, thereby promoting tenderness and juiciness. These improvements often lessen the need for technological interventions, contributing to more sustainable and minimally processed production systems.

Phytochemicals also play a pivotal role in flavor and aroma development by modulating lipid oxidation and enzymatic reactions. In milk, polyphenol interactions yield subtle nutty or fruity undertones that mask bitterness from lipid degradation and enhance creamy perceptions [31]. Volatile terpenes from phytochemical-rich forages impart floral and herbaceous aromas that distinguish organic or pasture-based milk, enhancing its sensory appeal [49]. In meat, similar processes occur as bioaccumulated plant secondary metabolites, such as β-caryophyllene and skatole, which generate “grassy” or “herbal” flavor profiles typical of grass-fed ruminants [2]. However, these sensory changes are not uniformly perceived as positive by all consumer segments. While premium and organic consumers often associate intense “grassy” or “herbal” notes with authenticity and naturalness, more conventional consumers may regard the same attributes as “barny” or overly strong, leading to lower overall liking scores [2,49,138]. Sensory studies on pasture-based and functional dairy products indicate that increases in green, herbaceous or animal-like notes can improve distinctiveness and perceived naturalness but may simultaneously decrease acceptability among individuals with low familiarity or low risk tolerance [48,138,139]. These findings highlight the need to calibrate phytochemical supply and feeding strategies to the target market and product category

Herbal supplements such as oregano and garlic further diversify sensory properties by introducing spicy or sulfurous volatiles that enhance perceived freshness and mask rancidity during storage [27]. In milk, interactions between volatile and polyphenol compounds can enhance flavor perception while allowing for reductions in fat, sugar, and salt without compromising overall acceptability [140]. Clarke et al. [48] demonstrated that milk derived from phytochemical-enriched diets exhibits distinct sensory signatures, where grass-fed milk scored higher for creamy color, while total mixed ration milk was associated with stronger hay-like flavor and lighter appearance. Volatile markers, including dimethyl sulfone, toluene, p-cresol, and ethyl esters, were identified as key drivers of these differences.

Consumer studies consistently support that sensory enhancement through phytochemical supplementation shapes product preference and market potential. Milk and meat from phytochemical-fed ruminants receive higher ratings for freshness, aftertaste, and overall flavour balance, primarily due to reduced oxidative off-notes [49,138]. Premium and organic consumers often favor the richer, natural profiles associated with phytochemical feeding, whereas conventional consumers may perceive these flavours as overly intense or unfamiliar [138]. Willingness-to-pay analyses further reveal nuanced consumer behaviour toward functional, phytochemical-enhanced dairy products. Zhen et al. [139] found that although most consumers resist paying premiums for functional milk, individuals with high-risk preferences exhibited significantly greater willingness-to-pay. Eye-tracking results indicated that visual attention to functional claims positively influenced purchase decisions, underscoring the importance of strategic communication in product marketing. Similarly, Lambiase et al. [141] reported that Spirulina-enriched mozzarella achieved higher sensory scores for brightness, butter odour, whey flavour, sweetness, and tenderness, yet acceptance was heavily dependent on providing consumers with information about Spirulina’s nutritional and functional benefits.

These findings demonstrate that phytochemicals enhance the sensory and nutritional value of animal-source foods and their market differentiation potential. Consumer demand for sustainable, natural, and health-promoting products highlights the need for integrated feeding strategies that optimize phytochemical inclusion. As Chalupa-Krebzdak et al. [142] emphasized, dietary interventions in livestock production can directly improve sensory quality and consumer satisfaction while advancing nutritional and functional value. Future research should therefore focus on elucidating the long-term impacts of phytochemical supplementation on milk and meat quality, with attention to bioactive stability, sensory evolution, and implications for functional food development in the modern dairy and livestock industries.

## 11. Antinutritional Effects of Dietary Phytochemicals on Ruminant Production and Product Quality

Excessive inclusion of dietary phytochemicals can negatively affect ruminant performance and product quality. High levels of polyphenols (>50 g/kg DM), particularly condensed and hydrolyzable tannins, reduce feed palatability due to their astringent properties, leading to decreased intake [143]. Condensed tannins inhibit microbial enzymes and damage cell walls, suppressing ruminal fermentation and slowing digestion. This contributes to gut fill, further limiting intake [143]. Beyond intake, polyphenols form stable complexes with dietary carbohydrates, lipids, proteins, and digestive enzymes, which reduces volatile fatty acid production in the rumen and limits the availability of fatty acids and amino acids for digestion and absorption in the small intestine [3]. This often results in reduced milk yield, protein, and fat content in dairy animals, while in meat-producing animals, weight gain, slaughter weight, and carcass weight decrease, leading to higher ultimate pH and darker, drier and less tender meat [3,144]. High polyphenol levels may also reduce meat tenderness by inhibiting calpains and collagenase matrix metalloproteinases, which correspondingly degrade myofibrillar proteins and intramuscular connective tissue [18]. In addition, excessive levels of polyphenol, especially those from aromatic plants and forages such as white clover and rye grass, can increase formation of methyl phenols (e.g., 4-methylphenol and 3-methylphenol), potent volatile compounds contributing to undesirable pastoral, grassy or herbal flavors in meat and milk [48,145].

Essential oils can cause feed aversion due to strong aroma and flavor, consequently lowering feed intake and weight gain [146]. Excessive supplementation may non-selectively inhibit rumen bacteria, reducing digestion rate and passage, which indirectly lowers intake and growth performance [3,146]. At elevated concentrations, essential oils may bypass ruminal degradation, form aromatic metabolites that accumulate in milk and meat, causing minty or herbal off flavour [147,148].

Saponins, glycosides with a bitter taste and foaming properties, impair palatability at concentrations above 10 g/kg DM [22,149]. In the rumen, high contents of saponins act as surfactants, stabilizing foam, which traps fermentation gases, inhibits eructation, and causes frothy bloat [150]. They can also disrupt protozoal cell membranes by binding cholesterol, causing lysis [151]. While this can benefit rumen fermentation, over-suppression of protozoa may destabilize ruminal microbiota, compromising microbial protein synthesis and volatile fatty acid production, ultimately reducing weight gain, milk yield and final body weight. High doses also irritate intestinal mucosa and inhibit fibrolytic bacteria, slowing nutrient digestion and absorption, reducing growth rate [151,152]. Evidence of the antinutritional effects of dietary saponins on the quality of ruminant edible products is limited. Given the inherent bitterness of saponins, it is hypothesized that their persistence in animal tissues may contribute to off-flavors in meat and milk, warranting systematic investigation.

Organo-sulphur compounds (>30 g/kg DM sulfate-S), such as glucosinolates in Allium and Brassica species, are converted to sulfides and hydrogen sulfide (H_2_S), which depress intake and fermentation [153,154]. Overexposure can cause scours, polioencephalomalacia, and rumen epithelial inflammation, impairing nutrient absorption [155]. Hydrolysis products like isothiocyanates and thiocyanates inhibit methanogens, reducing methane emissions, but excessive levels interfere with iodine uptake, inducing hypothyroidism and lowering feed efficiency, metabolic and growth rates [144,154]. High levels of organosulfur compounds in ruminant diets can lead to sulfur-derived volatiles transferring to edible products, causing garlic-like off-flavors [27,156].

Alkaloids are among the most toxic phytochemicals. Ergot alkaloids (>0.75 mg/kg DM), common in endophyte-infected tall fescue, cause fescue toxicosis by acting as vasoconstrictors. This reduces heat dissipation and blood flow to the digestive tract, impairing nutrient absorption [46]. High alkaloid intake also disrupts ruminal fermentation, reduces feed intake and growth [46], leading to lower milk solids and compromised carcass traits. Alkaloids, particularly pyrrolizidines from contaminated feed can accumulate in milk and meat, causing toxicity and altering its pH, color, and flavor [157,158]. Overall, excessive concentrations of phytochemicals can impair ruminant productivity and product quality, with the magnitude of impact influenced by dose, origin, and species. Gradual introduction, palatability monitoring, and adherence to recommended thresholds are essential to balance efficacy, animal performance, product quality and consumer satisfaction.

## 12. Emerging Technologies for Enhancing Phytochemical Bioavailability

The technological advancement of nanoencapsulation and rumen-protected formulations has emerged as a critical solution to overcome the inherent limitations of phytochemical bioavailability in ruminant nutrition systems. Phytochemicals, comprising flavonoids (9%), terpenoids (55%), and alkaloids (36%), face significant challenges, including poor water solubility, chemical instability, and extensive ruminal degradation that typically limits their absorption rates to below 20% [3]. Nanoencapsulation technologies address these limitations by creating protective barriers using food-grade materials such as lipids, proteins, and carbohydrates to form nano-emulsions, solid lipid nanoparticles, nanoliposomes, and biopolymer nanoparticles [159,160]. These delivery systems protect bioactive compounds from harsh environmental conditions, including heat, pressure, low pH, and digestive enzymes, while facilitating site-specific delivery in the rumen [59]. Lipid-based nanocarriers, particularly solid lipid nanoparticles composed of arachidic or stearic acids with Tween 60, have demonstrated remarkable resistance to ruminal digestion for up to 24 h while maintaining protection of their bioactive content from ruminal microbiota [161]. The encapsulation process involves trapping bioactive compounds within structurally engineered systems that provide thermodynamic and physical barriers against water vapor, oxygen, light, and enzymatic degradation, thereby improving the stability, bioavailability, and controlled release characteristics of phytochemicals at physiologically active sites [162,163]. In ruminant systems, most experimental applications of nanoencapsulation and rumen-protected delivery have so far focused on amino acids, lipids and selected essential oils, with encouraging but still limited in vivo evidence. Nanostructured carriers and rumen-protected matrices have been shown to increase post-ruminal delivery of limiting amino acids and improve milk protein yield in dairy cows, confirming that nanotechnology can enhance nutrient synchrony and efficiency of use under commercial conditions [6,45]. Similarly, encapsulated essential oils and tannins appear more effective than unprotected forms in modulating rumen fermentation and methanogenesis while minimising negative effects on intake and fibre digestibility, as highlighted by recent reviews on encapsulated essential oils and rumen-protected phytochemicals in ruminants [4,58,59]. These findings support the view that nanoencapsulation and related technologies can help translate the bioactivity of phytochemicals observed in vitro into consistent productive and environmental responses in vivo. Despite this promise, several practical and regulatory challenges may constrain the large-scale adoption of nano-delivery systems for phytochemicals in ruminant production. Reviews of food and feed nanotechnology emphasise careful assessment of the safety, environmental fate and potential bioaccumulation of nanocarriers, particularly inorganic or non-biodegradable materials, and to align new products with evolving regulatory frameworks and labelling requirements [4,16,43,58,102]. Moreover, consumer perception of “nano” technologies in the food chain is ambivalent: while encapsulation is often invisible at the product level, surveys indicate that some consumers express concerns about the safety and “naturalness” of nano-enabled foods and may prefer technologies framed as conventional microencapsulation or fat-encapsulation approaches [16,43,102]. Cost of production, patent protection and intellectual property issues further influence the commercial availability of nanoencapsulated phytogenic additives and may limit their use in high-value markets or intensive systems.

The practical application of nano-technologies in ruminant production systems has yielded measurable improvements in animal performance, product quality, and environmental sustainability. Controlled studies demonstrated that encapsulated phytochemicals, particularly tannins and essential oils, can significantly enhance milk production, with research showing increases from 36 to 37.9 kg/d in milk yield and improvements in energy-corrected milk from 37.1 to 39.7 kg/d [67]. These production benefits are associated with favorable modulation of rumen microbiota, including reductions in bacteria associated with low feed efficiency, such as *Selenomonas ruminantium* and *Streptococcus bovis*, while increasing total volatile fatty acid concentrations [8,67]. Encapsulated tannins demonstrate particular effectiveness in modulating ruminal biohydrogenation processes, leading to enhanced concentrations of beneficial fatty acids, including CLAS, 18:3n-3, and 18:sn-6 in both milk and meat products [52]. From an environmental perspective, encapsulated essential oils provide dual benefits by reducing methane emissions through their anti-methanogenic properties while maintaining or improving production efficiency [59]. The Technology Readiness Level for most encapsulation methods has progressed beyond TRL 6, indicating that functional prototypes are becoming commercially viable [164]. These technological advances support the development of sustainable ruminant production systems that simultaneously address production efficiency, product quality enhancement, and environmental impact mitigation through improved nutrient utilization and reduced greenhouse gas emissions [22,165].

Future research on emerging technologies for phytochemicals in ruminants should: (i) move beyond proof-of-concept trials to long-term in vivo studies under commercial conditions; (ii) systematically compare nanoencapsulated, microencapsulated and unprotected forms in terms of bioavailability, production and product quality responses as well as environmental health indicators; (iii) incorporate cost–benefit analyses and life cycle assessment to quantify economic and environmental trade-offs; and (iv) evaluate regulatory compliance and consumer acceptance of nano-enabled feed additives and the resultant ruminant products. Integrating nanotechnology with more conventional rumen-protection systems and locally available phytochemical sources could offer practical, scalable pathways to enhance the efficacy and consistency of phytochemical-based strategies for sustainable milk and meat production.

## 13. Processing Impact

Phytochemical enrichment markedly influences the processing behavior of animal products, modulating fermentation, coagulation, thermal stability, and oxidative properties across dairy and ruminant meat systems. In fermented dairy products, plant-derived bioactives accelerate fermentation kinetics, as olive leaf extract reduces yogurt fermentation time from 276 min in controls to 240–270 min depending on concentration [166]. Enhanced metabolic activity of *Streptococcus thermophilus* under phytochemical-rich conditions improves acidification and viability, while extracts from citrus peels, herbs, and fruit pomaces increase total phenolic content by 25–35% without compromising fermentation stability [166,167,168,169]. Apple and mulberry pomace further enhance water-holding capacity, consistency, and viscosity, reduce syneresis, and modify firmness during storage, although excessive extract concentrations may reduce apparent viscosity, necessitating careful optimization [169,170,171].

Cheese processing demonstrates similar interactions, as protein–polyphenol binding can delay coagulation at higher inclusion levels (>5% grape pomace), while low concentrations of pomegranate peel extract provide antimicrobial activity against *Escherichia coli*, *Staphylococcus aureus*, and *Salmonella* without inhibiting starter cultures [55,172]. Enriched cheeses exhibit improved oxidative stability, flavor development, and color retention during ripening, with flavonoid levels increasing to 40% [55,172,173]. Spray-drying of phytochemical-enriched milk similarly requires precise thermal control, with inlet temperatures of 150–180 °C retaining 70–80% of antioxidants while minimizing Maillard reactions and forming stable polyphenol-protein complexes, although higher phytochemical levels may reduce powder flowability, as indicated by Hausner ratios above 1.73 and Carr indices exceeding 42.29% [174,175,176].

In ruminant meat systems, polyphenols and flavonoids enhance oxidative stability, color retention, water-holding capacity, and microbial safety without modifying protein or fatty acid composition. Sesamol, ellagic acid, and olive leaf extract reduce TBARS and prevent oxymyoglobin oxidation in raw beef, while resveratrol and citroflavan-3-ol improve redness in lamb under high-oxygen modified atmosphere packaging [177,178]. Thermal processing further demonstrates the protective effects of grape seed extract, pine bark extract, and rosemary oleoresin, achieving 92–94% reductions in TBARS and hexanal, retaining redness, and suppressing pathogens including *E. coli* O157:H7, *Listeria monocytogenes*, *Salmonella typhimurium*, and *Aeromonas hydrophila* [179]. Beer-based marinades incorporating herbs, spices, terpenes, and sulfur compounds limit lipid oxidation and Maillard reaction products while enhancing terpene content and sensory appeal in grilled beef and moose [180].

Overall, phytochemical enrichment consistently improves oxidative stability, microbial safety, texture, and color across dairy and meat systems while preserving core nutritional components. Effectiveness varies with the type of extract, its concentration, and the food matrix: for example, in raw minced beef patties, lutein, sesamol, ellagic acid, and olive leaf extract impacted lipid oxidation, microbiological stability, and water-holding capacity [177], and in spray-dried skimmed goat’s milk powder enriched with grape-pomace seed extract, polyphenol–protein interactions improved antioxidant activity [176]. These findings underscore the potential of dietary phytochemicals as natural functional additives that enhance product quality and processing efficiency in modern animal-product systems. Nevertheless, the technological and sensory effects of phytochemical enrichment are non-linear and matrix-dependent. Excessive inclusion levels can accelerate acidification beyond optimal ranges, weaken gel networks, or increase syneresis in yoghurt, and may lead to coarse or brittle textures in cheese or spray-dried powders with poor flowability [169,170,171,174,175,176]. In meat systems, high doses of strongly coloured or bitter extracts can darken the product surface or introduce herbaceous and astringent notes that are not always preferred by consumers, despite clear gains in oxidative stability and microbial safety [177,178,179,180]. Recent reviews on dairy and meat fortification emphasise that plant extracts from by-products and herbs should therefore be optimised not only for antioxidant performance but also for their impact on colour, flavour, texture and overall acceptability, ideally using response-surface or multi-criteria optimisation approaches [173,179]. Integrating processing-level phytochemical enrichment with upstream feeding strategies offers a complementary pathway to design animal products that meet both technological and sensory targets within a clean-label framework.

## 14. Human Health and Functional Food Potential

The incorporation of dietary phytochemicals in ruminant feeds not only enhances animal production and sustainability but also elevates the nutritional profile of milk and meat, positioning these products as functional foods with significant implications for human health [2,179]. Functional foods, defined as those providing health benefits beyond basic nutrition, can be naturally enriched through ruminant diets rich in phytochemicals such as polyphenols, terpenoids, flavonoids, and carotenoids [2,3]. These bioactives transfer from forages to animal products via rumen metabolism and bioaccumulation, resulting in milk and meat with enhanced antioxidant, anti-inflammatory, and antimicrobial properties [39,47]. For instance, grass-fed systems accumulate higher levels of health-promoting phytonutrients in meat and milk than grain-fed counterparts, with metabolite profiles differing by up to 90% [2]. This enrichment supports human dietary needs, particularly in addressing micronutrient deficiencies and promoting wellness through everyday consumption of dairy and meat products [16,48]. It is, however, important to note that most of the evidence supporting these functional attributes derives from pre-clinical models, short-term feeding trials and observational studies, rather than from large, long-duration human interventions. Reviews on grass-fed meat and milk, as well as on nutritional interventions in ruminant products, consistently show favourable shifts in fatty acid profiles, antioxidant content and selected biomarkers, but also highlight substantial heterogeneity in study design, population, and background diet [3,10,181]. As a result, current data strongly support the biological plausibility of health benefits but remain insufficient to quantify long-term risk reduction at the population level.

Human health benefits from these enriched products are multifaceted, primarily stemming from their antioxidant and anti-inflammatory capacities. Polyphenols and carotenoids in grass-fed milk and meat act as potent antioxidants, scavenging reactive oxygen species (ROS) and reducing oxidative stress, which is implicated in aging and various pathologies [2]. Terpenoids, such as α-copaene and β-caryophyllene, exhibit anti-tumor and anti-bacterial effects, while phenols like chlorogenic acid and gallic acid provide cardioprotective and neuroprotective benefits [2]. In dairy, isoflavones (e.g., daidzein, genistein) from clover-based feeds transfer to milk, offering estrogenic properties that may alleviate menopausal symptoms and support bone health [47,48]. Similarly, CLAs, 1.5–3 times higher in grass-fed products, contributes to anti-adipogenic and anti-carcinogenic effects [2]. Consumption studies demonstrate elevated serum levels of CLAs, docosapentaenoic acid (DPA), eicosapentaenoic acid (EPA), α-tocopherol, and β-carotene in humans after ingesting grass-fed meat and milk, underscoring their bioavailability and potential for daily health maintenance [2,39].

Links to chronic disease prevention are particularly compelling, as phytochemical-enriched ruminant products modulate pathways associated with metabolic, cardiovascular, and oncogenic disorders [2,16,47]. For metabolic diseases like obesity and type 2 diabetes, grass-fed goat milk prevents insulin resistance and hepatic steatosis in high-fat diet mouse models by reducing body fat mass (19.1% vs. 35% in controls), improving glucose tolerance, and enhancing mitochondrial function through upregulated uncoupling protein-1 (UCP-1) and phosphorylated AMP-activated protein kinase (p-AMPK) [39]. Anti-inflammatory effects, such as downregulation of tumor necrosis factor-alpha (TNF-α) and interleukin-6 (IL-6), mitigate chronic inflammation linked to atherosclerosis and neurodegeneration [2,3]. Cardiovascular benefits include reduced low-density lipoprotein (LDL) oxidation and improved high-density lipoprotein (HDL) profiles, with carotenoids lowering type 2 diabetes risk [2,48].

Dairy innovations leveraging phytochemical enrichment represent a growing frontier, driven by sustainable feeding strategies that enhance product bioactivity without synthetic additives [2,58,102]. Innovations include pasture-based systems incorporating diverse forages (e.g., herbs, legumes) to produce “designer” milks with elevated isoflavones and CLAs, as seen in clover-fed bovine milk with 2–3 times higher daidzein and formononetin levels [48]. Supplementation with mixtures like curcuminoids and trans-cinnamaldehyde in dairy cows increases milk yield by 7.93% and shifts fermentation toward glucogenic profiles, potentially yielding functional milks with improved lactose (2.36% higher) and antioxidant stability [102]. Artisan cheeses from grazing goats show 2–5 times higher total polyphenol content (TPC) and antioxidant activity, with innovations in raw or minimally processed products preserving terpenes and flavonoids for enhanced shelf life and sensory appeal [39,58]. Epigenetic-focused innovations, such as maternal phytochemical diets altering offspring milk quality via gene expression changes, open avenues for transgenerational functional dairy [47]. Market-driven advancements, like “100% pasture-raised” labeling, capitalize on consumer demand for natural, health-promoting products, with U.S. grass-fed dairy showing a 10% compound annual growth rate [2]. Therefore, phytochemical-enriched ruminant milk and meat offer substantial potential as functional foods for chronic disease prevention, underpinned by antioxidant and epigenetic mechanisms. However, while animal models demonstrate efficacy, human intervention trials are needed to validate transfer efficiency and long-term benefits.

Future research should prioritise well-controlled human intervention trials using phytochemical-enriched milk and meat within realistic dietary patterns, with careful characterisation of dose, exposure duration and inter-individual variability. Such studies should integrate clinical outcomes, intermediate biomarkers (e.g., inflammation, lipid oxidation, insulin sensitivity) and metabolomic profiling to capture the contribution of ruminant-derived phytochemicals relative to other dietary sources [2,10,16,48]. In parallel, life cycle and socio-economic assessments are critical to ensure that functional products derived from grass-fed or phytochemical-enriched systems remain accessible and environmentally competitive [6,10]. Ultimately, phytochemical-enriched ruminant foods should be positioned not as stand-alone “cures” but as nutrient-dense components of diverse, balanced diets, where their added value lies in combining high-quality protein and essential micronutrients with a unique spectrum of bioactive compounds [2,10,45,48].

## 15. Regional and Global Perspectives

Applying phytochemicals in ruminant production represents a global research priority with significant regional variations in implementation and outcomes. Tedeschi et al. [3] emphasized that phytochemicals have been actively researched as feed additives worldwide, though academic and commercial interests in the technology are yet to be fully adopted. Global sustainability challenges underscore the importance of phytochemical applications across diverse production systems. Pulina et al. [6] noted that the expected increase in population and urbanization requires a long-term global strategy for more intensive and sustainable ruminant production. Provenza et al. [45] connects this to climate mitigation, identifying that regenerative agriculture practices involving phytochemically rich landscapes rank as the number one approach among 80 ways to alleviate climate change globally. Regional production systems demonstrate varying potential for phytochemical integration. Extensive ruminant production systems, particularly prevalent in Mediterranean and developing regions, naturally incorporate diverse phytochemicals through grazing, resulting in milk and cheese with enhanced nutritional profiles [43]. Therefore, pasture-based production systems represent a sustainable supplier of animal source foods worldwide, with plant diversity offering greater benefits than monocultures. At the same time, intensively managed ruminant systems in high-income regions often rely on conserved forages and concentrate-based diets with lower intrinsic phytochemical diversity, which may limit the spontaneous transfer of bioactive compounds to milk and meat unless targeted feed additives, shrubs or by-products are strategically included [17,19,182]. These contrasts illustrate that phytochemicals can be leveraged either through landscape-level plant diversity in extensive systems or via formulated phytogenic additives and tailored forage mixtures in more intensive systems [3,19].

Climate change presents universal challenges requiring regionally adapted solutions. Chauhan et al. [183] identified that climate change presents challenges for global animal agriculture, with oxidative stress affecting animal health globally. Ruminants contribute approximately 6% of global greenhouse gas emissions, making methane mitigation through phytochemicals a worldwide priority [4]. However, research intensity and outcomes vary significantly by region. Valenzuela-Grijalva et al. [69] observed that while phytochemical research in broilers and pigs shows consistent benefits, few studies have been conducted in ruminants with inconclusive results, suggesting regional research gaps. The global perspective reveals both opportunities and challenges. Vahmani et al. [181] noted that ruminant meat plays an important role in global food and nutrition security, while Ponnampalam et al. [10] emphasized that nutritional interventions are essential for achieving high-quality meat and milk products for diversified and competitive global markets. Dietary manipulation is the most effective and convenient way to optimize global production of milk and meat. Future success requires coordinated international research addressing regional variations in climate, production systems, and regulatory frameworks, adopting a multidisciplinary approach towards sustainable ruminant production that considers regional differences while addressing global sustainability challenges [184]. Integrating traditional knowledge with modern science across diverse geographical contexts remains essential for realizing phytochemicals’ full potential in global ruminant production systems.

## 16. Conclusions

Dietary phytochemicals offer a promising, though context-dependent, approach to enhancing ruminant health, production, product quality, and sustainability. These natural bioactive compounds, present in herbs, legumes, spices, and grasses, may support rumen fermentation, nutrient utilization, and animal health, while also showing potential to reduce methane emissions and nitrogen losses. Inclusion of phytochemicals in ruminant diets can improve milk and meat quality through higher levels of beneficial fatty acids, antioxidants, and vitamins, contributing to enhanced nutritional and sensory attributes. However, the magnitude and consistency of these effects are influenced by factors such as dose, diet composition, animal physiological status, and management conditions.

Phytochemical-rich feeds can support antimicrobial and anti-inflammatory functions, potentially reducing reliance on synthetic additives and antibiotics. From a sustainability perspective, these compounds may contribute to reducing the environmental footprint of livestock systems by improving feed efficiency and mitigating greenhouse gas emissions, though outcomes are not always uniform across studies. Technological advances, including nanoencapsulation and rumen-protected formulations, have improved bioavailability, yet responses remain variable and require careful dose optimization to ensure reproducibility without compromising animal welfare. Current evidence suggests phytochemicals primarily act as modulators of rumen microbiota, hydrogen flows, lipid metabolism, and host oxidative and inflammatory status. These effects often translate into incremental, biologically meaningful improvements in feed efficiency, product composition, shelf life, and sometimes animal robustness. Benefits tend to be more evident in milk and meat quality, antioxidant protection, and emission intensity than in absolute yield, particularly under pasture-based or low-input systems. Nonetheless, results may differ depending on the phytochemical source, formulation, and production context.

To fully realize their potential, further research is needed to establish standardized dose–response relationships, clarify molecular mechanisms, and identify region-specific applications that ensure economic and environmental feasibility. Future priorities include (i) multi-site, long-term in vivo trials assessing production, product quality, methane, and nitrogen outcomes; (ii) integration of multi-omics data to link rumen microbiome and metabolome shifts with animal performance and product traits; (iii) robust economic and life cycle assessments of phytochemical-rich diets; and (iv) evaluation of consumer acceptance and market opportunities for phytochemical-enriched milk and meat. Ultimately, dietary phytochemicals should be considered as part of a broader portfolio of climate-smart, health-oriented strategies. Their benefits are promising but dose-sensitive, context-dependent, and not always reproducible, underscoring the need for careful integration into feeding programs alongside genetics, grazing management, and feed processing innovations.

## Figures and Tables

**Table 1:** Dietary sources of phytochemicals for ruminants: bioactive compounds, functional properties, and applications in milk and meat production systems.

Plant Source	Phytochemical Type	Key Bioactive Properties	References
Alfalfa	Saponins	Reduces methane emissions, improves digestion, and nutrient utilization in milk and meat	[8]
Clove	Polyphenol	Antioxidant, antimicrobial; enhances immunity and reduces inflammation	[24]
Quebracho	Tannins	Protein protection, methane reduction, and improved feed efficiency	[25]
Natural and nature-identical feed additives	Essential oils (terpenes, phenylpropanoids) and standardized phytogenic compounds	Bio-preservative, antioxidant, anti-microbial activities; supports shelf life in dairy and meat products	[26]
Garlic	Organosulfur	Enhances immunity, antimethanogenic; reduces inflammation and improves cheese flavor	[27]
Turmeric	Curcuminoids	Antioxidant, anti-inflammatory; reduces aflatoxin residues in milk	[28]
Oregano	Carvacrol	Antimicrobial activity, antiviral and antifungal properties; modulates rumen fermentation	[29]
Grazing paddocks with *Poaceae* and dicotyledons	Terpene	Antimicrobial, anti-inflammatory, antioxidant, and antiallergic; elevates terpene content in milk	[30,31]
Tropical legumes (e.g., Leucaena)	Tannins and saponins	Methane mitigation, improved nitrogen utilization, and enhanced meat and milk fatty acid profiles	[32]
Willow fodder	Condensed tannins	Reduces methane emissions, supports blood composition, and is sustainable in grazing systems	[33]
Savory plant (Satureja khuzistanica)	Flavonoids and carvacrol	Improves rumen fermentation, nutrient digestibility, and boosts CLA and n-3 in milk	[34,35]
Mustard and cumin seeds	Essential oils and flavonoids	Enhances milk yield, fatty acid profile; improves feed utilization	[36]
Capsicum (chili)	Capsaicinoids	Increases milk fat, feed efficiency, and supports lactation under stress	[37]
Grape pomace and winery by-products	Polyphenols (tannins, anthocyanins, flavonols)	Source of polyphenols and tannins; may reduce rumen methane yield and modulate fermentation; improves antioxidant status and oxidative stability of milk and meat; valorises agro-industrial residues in circular production systems	[38,39,40,41]
Citrus by-products	flavonoids and essential oils (hesperidin, naringin, limonene)	Antioxidant and antimicrobial; citrus flavonoid extracts can improve growth performance, rumen development, carcass traits and meat quality in small ruminants, with potential as natural alternatives to antibiotic growth promoters	[42]

**Table 2:** Dose–Response Evidence for Dietary Phytochemicals in Ruminants.

Phytochemical Class	Source/ Example	Indicative Optimal Dose Range (Per kg DM)	Positive Effects (Production/ Quality/Sustainability)	Adverse Effects at High Dose (>Threshold)	Reference
Tannins (Condensed)	*Lotus* *pedunculatus*	20–40 g	Increased rumen escape protein, weight gain, and methane reduction	Indigestible complexes, enzyme inhibition, toxicity	[51]
Tannins (Hydrolyzable)	Pomegranate peel	50 g	Improved milk fatty acids, reduced nitrogen excretion, and rumen modulation	Liver stress, metabolic disruption	[80]
Saponins	Tea saponins	1–5 g	Protozoa reduction, enhanced citrulline/lanosterol metabolites, and nitrogen efficiency	Membrane damage, reduced bacterial populations	[65]
Essential Oils	Carvacrol-based	≤500 mg	Elevated ruminal pH, serum triglycerides reduction, and fiber digestibility	Increased ammonia, metabolic inefficiency	[20]
	Limonene-based	501–1000 mg	Propionate increase, ammonia decrease, milk yield enhancement	Palatability loss, reduced efficacy	[20]
Polyphenols/ Flavonoids	Daidzein	≤600 mg	Intake and milk production boost, antioxidant improvement	Production decline, bitterness effects	[21]
	Anthocyanin	401–700 mg	Intake improvement in moderate-concentrate diets, protein content rise	Milk yield decrease, adaptation issues	[21]
Organosulfur Compounds	Garlic-derived	Limited data; ~200–500 mg	Potential antimicrobial activity, fermentation modulation	Flavor transfer to products, intake reduction	[3]

Note: Indicative optimal ranges are based on in vivo studies and recent meta-analyses on tannins, saponins, essential oils and flavonoids in ruminant diets; actual safe levels depend on species, physiological stage, basal diet and additive formulation.

**Table 3:** Effects of major phytochemical classes on milk yield and composition and on meat performance and quality in ruminant livestock.

Phytochemical Type	Effect on Milk Yield	Effect on Milk Composition	Effect on Meat Yield/Performance	Effect on Meat Composition/Quality	Citations
Saponins	Variable; can increase milk yield in some contexts	Can increase milk fat and protein when combined with essential oils compared with monensin	Species- and source-dependent; some extracts improve average daily gain	Modulate rumen fermentation and nutrient use; potential to improve carcass traits, though responses are inconsistent	[22,95]
Tannins	Generally little or no effect on corrected milk yield; small increases in some models	Modulate N use; can alter milk FA profile, increasing n-3, CLA and vaccenic acid	Do not consistently increase growth rate, but may improve protein utilization	Improve meat FA profile (↑ 18:3n-3, CLA, trans-11 18:1; ↓ some SFA) and can enhance flavour and reduce off-flavours	[52,96,97]
Essential oils	Often increase or maintain milk yield with improved feed efficiency	↑ milk fat or protein in some trials; ↓ SFA and ↑ MUFA/PUFA with certain blends	Limited direct data on carcass yield; some EO–polyphenol blends improve performance	Improve oxidative stability and may favourably modify FA profile and shelf-life of meat	[35,97,98,99,100,101,102]
Flavonoids	↑ milk production, fat and protein content in meta-analysis	↓ oxidative markers; improved antioxidant status; better milk fatty acid profile	↑ daily weight gain and feed efficiency in beef cattle	↓ shear force (more tender), ↓ malondialdehyde, improved color (less yellowness), enhanced antioxidant status of meat	[21,97,100]
Carvacrol (phenolic monoterpene)	In mixtures, can support higher milk fat and protein; contributes to improved FA profile (↑ oleic, unsaturated FA)	↓ milk SFA and ↑ MUFA/PUFA and CLA when supplied via savory or EO blends	Not primarily tested for carcass yield; may contribute to better growth via improved digestion	Antioxidant and antimicrobial activity contributes to improved meat oxidative stability and possibly FA profile	[2,35,97,98,100,101]
Terpenes	Do not directly increase milk yield but increase terpene content in milk from diverse pastures	↑ mono- and sesquiterpenes and other phytonutrients with antioxidant/anti-inflammatory properties in milk	Grazing diverse, terpene-rich pastures can support normal growth while enriching meat in terpenoids	↑ terpenoids and other phytonutrients in meat, contributing to antioxidant activity and potential health benefits	[2,100]
Capsaicinoids (phenylpropanoids)	As part of EO blends, can increase milk yield and fat concentration and improve feed efficiency	↓ SFA and ↑ unsaturated FA and CLA in milk fat in some EO-capsaicin products	May improve performance via better rumen fermentation and antioxidant/anti-inflammatory effects	Contribute to antioxidant, anti-inflammatory protection, potentially improving meat oxidative stability and colour	[35,97,99,100,101,102]

Abbreviations: CLA, conjugated linoleic acid; MUFA, monounsaturated fatty acids; PUFA, polyunsaturated fatty acids; SFA, saturated fatty acids; EO, essential oils; FA, fatty acids). Arrow notation: ↑ indicates an increase; ↓ indicates a decrease.

## Data Availability

No new data were created or analyzed in this study. Data sharing is not applicable to this article.
